# Peracetic acid treatment of squid eggs infected with parasitic copepod (*Ikanecator primus* gen. et sp. nov.)

**DOI:** 10.1038/s41598-024-65290-z

**Published:** 2024-06-24

**Authors:** Mehmet Arif Zoral, Zdenek Lajbner, Lucia Zifcakova, Jonathan Miller

**Affiliations:** Physics and Biology Unit, Okinawa Institute of Science and Technology Graduate University (OIST), 1919-1 Tancha, Onna-Son, Okinawa, 904-0945 Japan

**Keywords:** Cephalopod, *Sepioteuthis*, Copepod, *Ikanecator primus*, Peracetic acid, Parasitology, Pathogens, Antiparasitic agents

## Abstract

Having been successfully bred in semi-intensive and intensive aquaculture systems, oval squids of the *Sepioteuthis lessoniana* species complex are emerging as promising candidates for research and industry. Nevertheless, information about pathogens and diseases that may affect squid aquaculture remains sparse. In this study, we identify new parasitic copepod species that causes squid mortality and decreases squid hatching rates, and we also offer a solution to eliminate the pathogen during incubation of squid eggs. The newly discovered copepod *Ikanecator primus* gen. et sp. nov. was identified on oval squid eggs for the first time using both morphological and molecular diagnostic markers. In the genomes of the copepod and associated microbiome, we identified multiple genes for enzymes involved in cephalopod eggshell degradation in genomes of the copepod and associated microbiome. Furthermore, we conducted experiments to assess efficacy of peracetic acid in inhibiting the *I. primus* gen. et sp. nov. both in vitro and in vivo using immersion treatment. We established that a 2-min exposure to a concentration of 250 μl/L of peracetic acid containing product (PAA-product; 35 mg/L PAA and 15 mg/L H_2_O_2_) inhibited the development of nauplii in vitro. All parasites exposed to a concentration of 500 μl/L of PAA-product (70 mg/L PAA and 30 mg/L H_2_O_2_) were eliminated within two minutes. On top of this, the immersion treatment with 500 μl/L of PAA-product (70 mg/L PAA and 30 mg/L H_2_O_2_) improved survival of squid embryos and increased size of squid hatchlings compared with control and the immersion treatment with 125 μl/L of PAA-product (17.5 mg/L PAA and 7.5 mg/L H_2_O_2_) and the immersion treatment with 250 μl/L of PAA-product (35 mg/L PAA and 15 mg/L H_2_O_2_). These findings suggest that PAA holds a great potential as inhibitor and controller of parasitic copepod infections and for overall health management in cephalopod culture.

## Introduction

Human population is growing very rapidly, and it becomes difficult to meet its protein demand^1^. Thus, it is important to look for alternative species that are suitable for intensive aquaculture. Cephalopods, such as squids, are promising not only as animal models in various kinds of fundamental research, but also as alternative aquaculture animals because they have a high growth rate^2,3^, high protein content^3^, and high market value^2,3^. Additionally, they are reaching market size in less than one year which makes them a preferable option for many fisheries^3,4^. Therefore, in recent years, cephalopod aquaculture attracts increased attention of commercial subjects and becomes an important research topic itself.

Oval squid, *Sepioteuthis lessoniana* Férussac in Lesson, 1830, forms a demersal neritic species complex which inhabits coral reefs and seagrass beds in Indo-West Pacific Ocean, and it is now spreading also in the Mediterranean^5^. This species complex consists of three members that have been traditionally and routinely recognized by fishermen in Okinawa, Japan, but a detailed taxonomic revision is missing, and their morphological, molecular, and behavioral characterizations are incomplete^6–11^. Oval squids have been cultured successfully under artificial and/or semi-artificial conditions^5^.

This study has focused mainly on *S. lessoniana* sp.1, also called “Aka-ika” or red-squid, and the immersion treatment has been tested also on eggs of the other two members of the oval squid species complex: *S. lessoniana* sp.2, also called “Shiro-ika” or white-squid and *S. lessoniana* sp.3, also called “Kua-ika” or small-squid^8^. Pathogen screening and disease management methods for squid culturing are still in the early research phase. A good health management program in aquaculture helps the business to develop economically by preventing the loss of eggs, hatchlings and broodstock^12^. Therefore, it is crucial to extend new research topics in health management to sustainable systems for disease control and animal welfare improvement.

Small aquatic crustaceans, commonly known as copepods, inhabit a wide range of salinities, from fresh water to hypersaline conditions. They can be free-living, symbiotic, or internal or external parasites of fish or other aquatic animals^13,14^. Parasitic copepods feed on blood, mucus, and epidermal tissue of their host^15^. Increasing aquaculture activity and density provide better physicochemical, biotic, and epidemiological conditions for parasitic copepods^16,17^. Infestations can lead to low egg quality and high mortality rates, resulting in economic losses in fish hatcheries^16,18^.

Peracetic acid (PAA; CH_3_CO_3_H) is widely used as therapeutic agent against bacterial, viral, fungal, and parasitic infections in a variety of fields, including aquaculture, veterinary medicine, agriculture, medical science, and food processing^19–24^. In addition, PAA can be considered an eco-friendly agent since it degrades into harmless residues (acetate, water, and carbon dioxide). Few putative negligible adverse effects have been observed on fish and shrimp and cephalopod data are deficient^25,26^. One of the commercial solutions for PAA is an acidic quaternary equilibrium mixture of hydrogen peroxide (H_2_O_2_), acetic acid, and water. It occurs at room temperature in aqueous solution in equilibrium as ${\text{CH}}_{{3}} {\text{COOH }} + {\text{ H}}_{{2}} {\text{O}}_{{2}} \leftrightarrow {\text{ CH}}_{{3}} {\text{CO}}_{{3}} {\text{H }} + {\text{ H}}_{{2}} {\text{O}}.$

Here, we describe a new copepod genus and species that infects squid eggs. We also evaluate an effect of a peracetic acid-containing product (PAA-product) on the parasitic copepod infection on squid eggs and a potential application of PAA as therapeutic agent in squid culture.

## Materials and methods

### Source of cephalopod eggs and parasites

Squid eggs were obtained from the squid breeding program of Physics and Biology Unit approved by the Okinawa Institute of Science and Technology Graduate University Animal Welfare Committee (OIST; ACUP 2020–020 and ACUP 2023–036) at the Marine Science Station of Okinawa Graduate University of Science and Technology (MSS) in Okinawa, Japan, which routinely reproduces oval squid and kept one inbred line for 10 consecutive generations, patent application numbers JP2024-038,284 and JP2024-039,335. In addition, this study is reported in accordance with ARRIVE guidelines (https://arriveguidelines.org). All methods were carried out in accordance with the guidelines and regulations for this study, and no violations of ethical conduct were reported.

Since it has been established in 2016, MSS, which has a flow-through water system, is constantly threatened by external pathogens. Therefore, we are consistently monitoring pathogens and health status of our experimental animals, including squid eggs. We recorded a constant year-round presence of copepods within the squid eggs incubating environment at our facility.

All squid eggs used in each experiment of this study were always obtained from a single clutch laid in one day by a single female per squid species to eliminate any possible differences between mothers and clutches in size of yolk, composition of egg cases, or keeping environment and other experimental conditions. The eggs were examined for parasite infection under a dissecting microscope (Leica, S9D, Wetzlar, Germany) and a light microscope (Leica, DM2500 LED, Wetzlar, Germany). While conducting our microscopic investigation, we applied a brief seawater bath to the eggs, followed by occasional supplementary exposures to minuscule amounts of fresh seawater to retain their moisture balance. During the incubation period of squid eggs, parasitic infections were observed in all stages of developing embryos^27^ and 100% parasitic prevalence was recorded. Copepods were collected gently from egg’s surface, using demineralized water bath and/or fine needles for morphological analysis and molecular identification. Several substances such as liquid nitrogen, glutaraldehyde, methanol, ethanol, and formaldehyde were used for copepod preservation for various purposes described below.

### Light and scanning electron microscopy

Infected squid egg cases within 24 h after hatching and individual parasites were placed on microscope slides in a small drop of seawater, and a coverslip was placed over the specimen with pressure for observation of their basic morphology and behavior. Some of the copepods were preserved in 10% formalin or 99% ethanol at room temperature so they could be further examined later. Some fresh parasites were mounted using medium with glycerin jelly for light microscopy for long term observation. In addition, some parasites were kept in 2% glutaraldehyde in PBS 1X at pH 7.4 for scanning electron microscope examination for morphology. The samples were washed 3 times with phosphate buffered saline (1X, pH 7.4) and post-fixed in buffered 1% osmium tetroxide. The samples were dehydrated through a graded ethanol series and dried on freeze dry. Final stage, the samples were coated with gold-silver alloy using Multifunction vacuum deposition equipment (VE3030CVD, Tokyo, Japan). Observations were completed using JEOL, JSM-7900F scanning electron microscope.

### DNA extraction and sequencing of parasitic copepod

Molecular identification was performed as follows: copepods were frozen and stored in liquid nitrogen. The specimens were processed as pooled sample using the Zymo Xpedition Tissue and Insect DNA MiniPrep kit to extract genomic DNA (Zymo Research Corp., Irvine, California) by OIST SQC. The manufacturer's instructions were largely followed for DNA extraction, with some key modifications. Samples were incubated at 55 °C in storage/stabilization buffer with 20 μl (20 mg/mL) proteinase K overnight. Beta-mercaptoethanol was also added to the genomic lysis buffer, raising its concentration to 0.5% before it was added to the BashingBead Lysis supernatant.

Shotgun libraries were prepared using NEBNext® Ultra™ II FS DNA PCR-free Library Prep Kit for Illumina® (New England BioLabs Inc., Ipswich, UK). The libraries were enriched by performing 5 cycles of PCR, as outlined in the manufacturer’s protocol, and sequenced at 300 bp paired-end on MiSeq v3.

The datasets including raw sequencing data and annotated DNA fragments generated and analysed during the current study are available in the NCBI Genbank repository, project number PRJNA1065100 (Supplementary Table 1). The Nextflow bacass/2.1.0 pipeline that implements FastP and FastQC was used to remove low quality reads. We screened a total of 17,680,144 reads, of which 99.82% were used for further metagenomic assembly. SPAdes/3.15.5 + dfsg-2 assembly was run with default parameters. Assembly statistics were obtained using Quast/5.2.0. Repetitive sequences were predicted using RepeatModeler and masked using RepeatMasker software within Dfam TE Tools Container/1.87.

Taxonomy and function were assigned to scaffolds by ncbi-blast/2.10.0 + with Diamond blast/2022-07 database. Additionally, for taxonomy overview, Kraken2 was used with k2_standard_8gb_20210517 database. dbCAN3 server was used to find carbohydrate active enzymes and auxiliary enzymes. dbCAN3 uses the enzyme classification system from Carbohydrate Active EnZymes database (www.cazy.org). We have selected scaffolds annotated as Arthropoda and use them to check the copepod genome completeness with BUSCO software and arthropoda_odb10 database^28^.

Raw reads (available in Genbank under project number SRR27696250, SRR27696251 and PRJNA1065100) were imported also into Geneious Prime (version 2023.2) for downstream analysis and mapped to reference sequences (18S, COI and mitogenome from related species, *Amphiascoides atopus*), visually inspected, and used to construct phylogenetic trees in RAxML v. 8.2.11^29^ implemented in the Geneious. Accession numbers of DNA sequences used in this study are listed in the Supplementary Table 1. DNA sequences coding for the small subunit ribosomal RNA (18S rRNA) and the cytochrome oxidase I (COI) containing mitogenomic sequence are available in NCBI Genbank (PRJNA1065100) under accession numbers PP196486 and PP163433 respectively (Supplementary Table 1).

### Antiparasitic effects of PAA-product in vitro

The PAA-product was acquired from Mitsubishi Gas Chemical Company, Inc., located in Tokyo, Japan. This solution contains 14% peracetic acid (PAA), 6% hydrogen peroxide (H_2_O_2_), 40–50% acetic acid, 30–40% water, and < 1% 1-Hydroxyethlidene-1,1-diphosphonic acid.

For this experiment, nauplii and adult copepods were observed on naturally infected red-squid egg cases within 24 h after squid hatch (Supplementary Fig. 1, Supplementary Video 1 and Supplementary Video 2). Adult copepods were collected using fine needles and placed in 6-well culture plates Costar 3516 (20 copepods /well). The plates contained PAA-product diluted with filtered seawater at concentrations of filtered seawater to final concentrations of 125 μl/L (17.5 mg/L PAA and 7.5 mg/L H_2_O_2_), 250 μl/L (35 mg/L PAA and 15 mg/L H_2_O_2_), 500 μl/L (70 mg/L PAA and 30 mg/L H_2_O_2_), 1000 μl/L (140 mg/L PAA and 60 mg/L H_2_O_2_) at 23 °C. There was also a control group of the plates without any treatment but in the same volume at 23 °C. This was done with three replicates per treatment. The copepods were then observed under a dissection microscope to record the time of death. Death was defined as ceasing or lack of movement. The time of death for each adult parasite was recorded until 100% of copepods in the wells cease of movement.

The same procedure and conditions were applied to 20 adult female copepods with their eggs (20 copepods /well) except that, the copepods were exposed to different amounts of PAA as follows: 125 μl/L (17.5 mg/L PAA and 7.5 mg/L H_2_O_2_), 250 μl/L (35 mg/L PAA and 15 mg/L H_2_O_2_), 500 μl/L (70 mg/L PAA and 30 mg/L H_2_O_2_), and 1000 μl/L (140 mg/L PAA and 60 mg/L H_2_O_2_) at 23 °C for 2 min. After the treatment, eggs were transferred to new 6-well plates containing filtered seawater and were incubated at 24 °C for 18 days until they hatched. Nauplii in each treatment group were recorded.

### Acute toxicity test on squid embryos before PAA-product immersion treatment application

The Trimmed Spearman-Karber method^30^ with 95% confidence intervals was used to determine the LC50 of PAA-product at 2 min. Total of 240 normally developing red-squid embryos from a single clutch of eggs laid by single female at the same day were used in three replicate acute toxicity tests. Stages of embryos were 23–24^27^. Red-squid strings containing 10 eggs with embryos were placed in each aerated 10 L glass aquaria together with PAA-product at 125 μl/L (17.5 mg/L PAA and 7.5 mg/L H_2_O_2_), 250 μl/L (35 mg/L PAA and 15 mg/L H_2_O_2_), 500 μl/L (70 mg/L PAA and 30 mg/L H_2_O_2_), 1000 μl/L (140 mg/L PAA and 60 mg/L H_2_O_2_), 2000 μl/L (280 mg/L PAA and 120 mg/L H_2_O_2_), 4000 μl/L (560 mg/L PAA and 240 mg/L H_2_O_2_) and the control treatments that did not contain PAA-product. Each string of 10 eggs represents a separate biological replicate. Treatments were within a range where 100% survival was expected in the lowest treatment and 100% mortality was expected in the highest treatment. Water temperature, oxygen and pH in the aquariums were 23 °C, 8 ppm and 8.2 respectively. After 2 min immersion application, eggs from all treatment groups were transferred and acclimated in plastic baskets (each treatment group: 3 strings = 30 eggs per basket) in one large tank with constant flow of filtered new water (100% water change per 1 h), at > 70% O_2_ saturation, pH 8.2 and temperature at around 23 °C, and monitored until they hatched about 2 weeks after the immersion. Embryos that hatched (see section “Hatching rate of squid eggs and hatchling size”) and maintained signs of life were recorded as living, whereas embryos that exhibited no signs of life (i.e. no movement) or failed to hatch were recorded as mortalities.

### Immersion treatment with PAA-product

#### Experimental design

The immersion treatment was conducted in a closed system that is made of a single glass aquarium. Squid eggs from a single clutch of a single female per each species (*S. lessoniana* sp. 1,2,3) were placed in 10 L glass aerated aquarium using air stone at 23 °C (temperature equal to the incubator at that time) and exposed to four different concentrations of PAA-product; 125 μl/L (17.5 mg/L PAA and 7.5 mg/L H_2_O_2_), 250 μl/L (35 mg/L PAA and 15 mg/L H_2_O_2_), 500 μl/L (70 mg/L PAA and 30 mg/L H_2_O_2_), 1000 μl/L (140 mg/L PAA and 60 mg/L H_2_O_2_) and the control (without PAA) for 2 min with aeration. Eggs were assessed for mortality until hatched using visual observations described above to determine the LC_50_ value. At the end of experiment, eggs were transferred and acclimated in plastic baskets (30 eggs per basket) with constant flow of filtered new water (100% water change per 1 h), at > 70% O_2_ saturation, pH 8.2 and temperature at around 23 °C. Eggs were kept until they hatched about 2 weeks after the treatment. At the end of the immersion treatment experiments, adult parasites were observed on squid eggs to calculate prevalence (percent of eggs containing at least one parasite) between treatment groups and the control (Supplementary Videos 1, 2).

#### Hatching rate of squid eggs and hatchling size

The hatching rate [percentage hatchability = (number of live squid hatchlings /number of all eggs) × 100] of each experimental group was examined and recorded after all surviving embryos hatched. We measured the total mantle length (cm) of squid in videos recorded within 24 h post hatching, using the ImageJ software. Each examined square surface was adjusted to the magnification of the image and an attempt was made to count all possible areas.

#### Monitoring of hatchlings

Within 24 h after hatching, hatchlings from 500 μl/L (70 mg/L PAA and 30 mg/L H_2_O_2_) PAA-product treatment (the minimum effective dose against copepods and a safe dose for squid eggs) and control groups were transferred to 60 L glass tanks (W 50 cm; L 40 cm; H 40 cm; 45 hatchlings /tank) and acclimated with constant flow of filtered new water (100% water change per 1 h), at > 70% O_2_ saturation, pH 8.2 and temperature at around 23 °C. They were kept for 4 weeks to observe any abnormal behavior. Twelve animals from the treatment group were raised to maturation. Their offspring has been exposed to the same treatment by 500 μl/L (70 mg/L PAA and 30 mg/L H_2_O_2_) PAA-product and equivalent keeping conditions.

### Statistical analysis

All results of in vitro, acute toxicity assay and immersion treatment results including hatchlings size and survival rate were compared by ordinary one-way ANOVA with Bonferroni’s and Tukey’s multiple comparisons test using GraphPad Prism software version 10 (GraphPad Software, San Diego, CA, USA). The probability level for statistical significance was set to *p* < 0.05 throughout the study.

### Ethical approval

All applicable international, national, and institutional guidelines for animal testing, animal care, and the use of animals were followed and with the oversight of the Okinawa Institute of Science and Technology Graduate University Animal Welfare Committee (OIST; ACUP 2020-298 and 2023-036). All the OIST animal facilities have been fully accredited at AAALAC International since July 2014. In addition, the research adhered to the ARRIVE guidelines introduced to improve the quality of methodological reporting of experimental animal research. These guidelines, available on the website https://arriveguidelines.org, were strictly followed throughout the study and ensure compliance with ethical conduct and regulatory standards.

## Results

### *Ikanecator* Zoral and Lajbner, gen. nov.

Order: Harpacticoida Sars, 1903.

Family: Miraciidae Dana, 1846.

Genus: *Ikanecator* gen. nov. Zoral and Lajbner, 2024.

Species*: Ikanecator primus* gen. et sp. nov., Zoral and Lajbner, 2024.

Type host: *Sepioteuthis lessoniana* sp.1, *Sepioteuthis lessoniana* sp.2, *Sepioteuthis lessoniana* sp.3, *Sepia pharaonis*, *Sepia latimanus*

Locality: Okinawa Island, Japan.

Site of infection: Squid and cuttlefish eggs.

Prevalence: 100% (all examined squid and cuttlefish eggs in the MSS were infected with *Ikanecator primus* gen. et sp. nov.)

**Figure 1:**
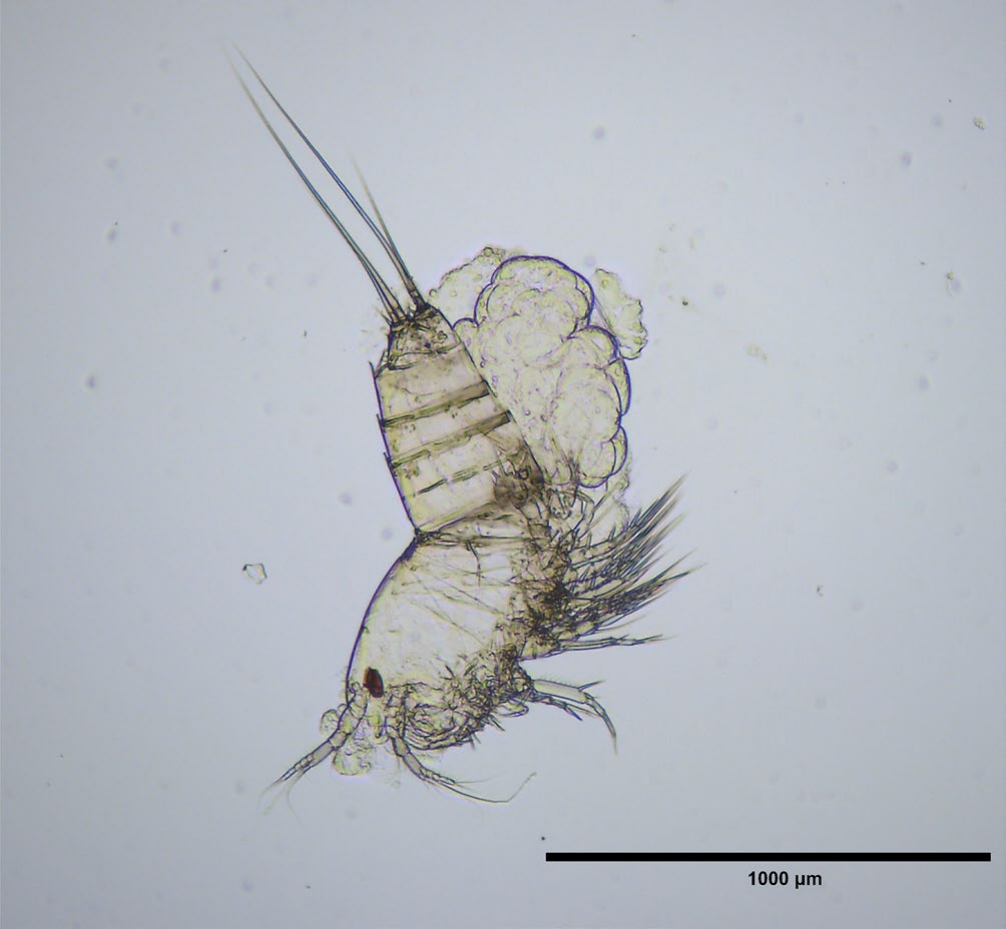
*Ikanecator primus* gen. et sp. nov., holotype, female (NMP P6E 5485).

Type material: Holotype: adult female, 1255.8 μm body length, collected by M. A. Zoral on August 8, 2023, on red-squid eggs in the OIST Marine Science Station, Onna, Okinawa Island, Japan (26°29′N; 127°51′E) was prepared as semi-permanent glycerine jelly slide-mount (Fig. 1) and deposited in the National Museum of the Czech Republic (NMP P6E 5485). Paratypes collected by M. A. Zoral on 2nd May, 2023, in the OIST Marine Science Station, were preserved in 2% glutaraldehyde for electron microscopy and deposited in the National Museum of the Czech Republic (11 individuals in NMP P6E 5486 and 14 individuals in NMP P6E 5487). Paratypes collected by M. A. Zoral on 6th November, 2023, in the OIST Marine Science Station, are preserved in 10% formalin or 99% ethanol and deposited in the National Museum of the Czech Republic (NMP P6E 5482: 4 females and 8 males in formalin, and NMP P6E 5483 4 females and 6 males in ethanol), Museum National d’Histoire Naturelle of France (MNHN-IU-2022-450: 4 females and 6 males in formalin; MNHN-IU-2022-451: 5 females and 5 males in ethanol) and Okinawa Prefectural Museum in Japan (OPM-Cr-1000227: a single female in formalin and OPM-Cr-1000228; 5 females and 5 males in ethanol).

Reference sequences: The raw reads are available in Genbank, the United States National Library of Medicine, National Center for Biotechnology Information, annotated database of all publicly available DNA sequences (SRR27696250, SRR27696251 and PRJNA1065100). DNA sequences coding for the small subunit ribosomal RNA (18S rRNA) and the cytochrome oxidase I (COI) containing mitogenomic sequence are also available in Genbank (PRJNA1065100) under accession numbers PP196486 and PP163433 respectively (Supplementary Table 1) that were used for phylogenetic tree reconstructions.

Etymology: The prefix ‘ika’ from the Japanese, refers to squid and cuttlefish common name and the suffix ‘necator’ from the Latin refers to killer or murderer. Gender masculine. The specific epithet of the species *Ikanecator primus* refers to its status as the first species of the genus to be described and the first documented copepod parasite on cephalopod eggs, known to us. The Latin word "primus", which means "first", is combined with the genus name to indicate its uniqueness as the type species.

Diagnosis (based on *Ikanecator primus* gen. et sp. nov.): The newly discovered genus, *Ikanecator* gen. nov., is a part of the Miraciidae family, Harpacticoida (Order), Copepoda (Class) and it is recognized by a combination of characters. Its body shape is fusiform. The rostrum, which is not fused to the cephalothorax, is elongated and is almost as long as the first, second, and third antennulary segments combined. It has a subdistal sensilla on each side that issues at the third quarter of its length. The pro- and urosomites do not have extensions and there is a flexible unsclerotized cuticle between the prosome and urosome, with the latter being more evident in the male. The female genital double-somite (which consists of the second and third urosomites) is completely fused ventrally, with a dorsolateral cuticular rib marking the former division between the somites. The anal somite is trapezoidal and without an anal operculum. The caudal rami is 0.8 times shorter in length compared to the entire body of the parasite unornamented, with a slender tubes pore ventrally and four setae. The female antenna is comprised of eight segments, with the fourth segment featuring a fused aesthetasc and seta. The male antennule shows sexual dimorphism and is made up of 11 segments, with the aesthetasc and seta fused at the base on the sixth segment. The antenna has an exopod (EXP) with three segments. The mandibular, has a one-segmented endopod (ENP) and a two-segmented EXP. The mandibular palp biramous, with a one-segmented ENP and a two-segmented EXP. The maxillule is biramous, with one-segmented rami. The maxilla has three endites. The maxilliped consists of four segments, with the endopod comprising a single segment carrying a claw and three setae. First (P1), second (P2), third (P3) and fourth (P4) swimming leg have three-segmented rami. P1 and P2 EXP is shorter than ENP. P3 EXP and P3 ENP are roughly the same length, with the ENP being shorter than the EXP at P4. Male P1 has a curved extension in basis which indicates sexual dimorphism. Male P2 is sexually dimorphic and consists of two segments. The first segment has a single inner seta, while the second segment has two inner setae, one of which is almost straight and located subdistally. The other two setae arise from an elongate cylindrical extension fused to the base of the segment. The second segment also has a strong outer subdistal spine that tapers distally. Male P2-P4 EXP1 is without one inner seta, while EXP2 is with one inner seta. P2-P4 ENP1 has one inner seta. P2 ENP1 has unguiform. P3-P4 ENP2 has one inner seta, one unguiform, inner distal seta of EXP3 visibly shorter than other elements of same segment. In the female, the P5 EXP separates, whereas in the male sex, the baseoendopods are fused medially. The P5 rami are distinct in both sexes. P5 EXP has six setae in both sexes, with the ones in the female sex being whip-like, and the two outer medial elements in the male transformed into short spines. In the female sex, the P5 endopodal lobe has five setae, whereas in the male sex, there are two setae. The armature formula of legs is given in Supplementary Table 2 for reference.

Sexual dimorphism is evident in the male antennule, P1 basis, P2 ENP, P5 and in the genital and third urosomites. Males exhibit these structures which are differentiated when compared to females.

Apomorphies of *Ikanecator* gen. nov.: This new genus can be recognized by (i) The length of ENP1 is either equal to or shorter than the length of EXP for both sexes, unlike in the closely related genera Amphiascoides, Paramphiascella and Robertgurneya; (ii) The P1 ENP2 has a distinct shape, resembling a wrench tool, which sets it apart from the typical shape of other endopods in both sexes. In addition, ENP2 has a distinctive hole far from the middle center close to the ENP3 area.

### *Ikanecator primus* Zoral and Lajbner, gen. et sp. nov. morphology

**Figure 2:**
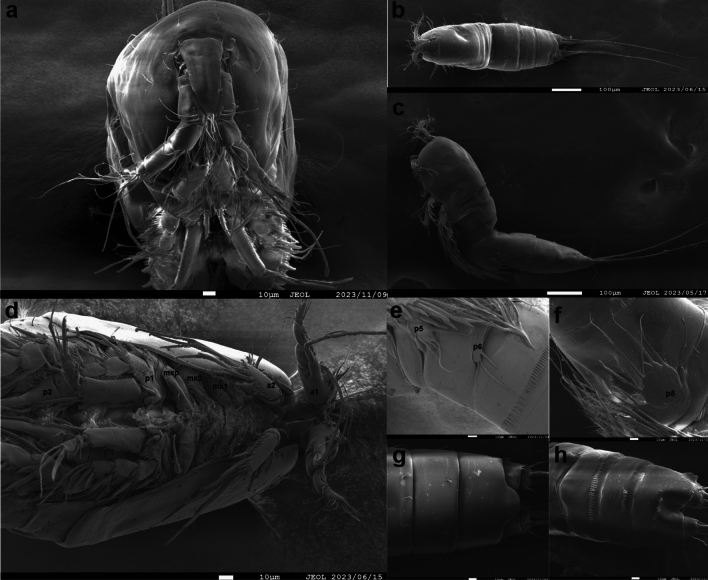
*Ikanecator primus* gen. et sp. nov., paratype. (**a**) The female cephalosome in front view, rostrum and antennule (NMP P6E 5486). (**b**) The female dorsal view (NMP P6E 5486). (**c**) The female left lateral view (NMP P6E 5486). (**d**) The male, antennule (a1), antenna (a2), maxillule (mx1), maxilla (mx2), maxilliped (mxp), swimming leg 1 (p1) and swimming leg 2 (p2) (NMP P6E 5486). (**e**) The male, swimming leg 5 (p5) and swimming leg 6 (p6) in ventral view (NMP P6E 5487). (**f**) The female, genital somite and swimming leg 5 (p5) in ventral view (NMP P6E 5486). (**g**) The female, urosome in dorsal view (NMP P6E 5487). (**h**) The male, urosome in ventral view (NMP P6E 5487).

**Figure 3:**
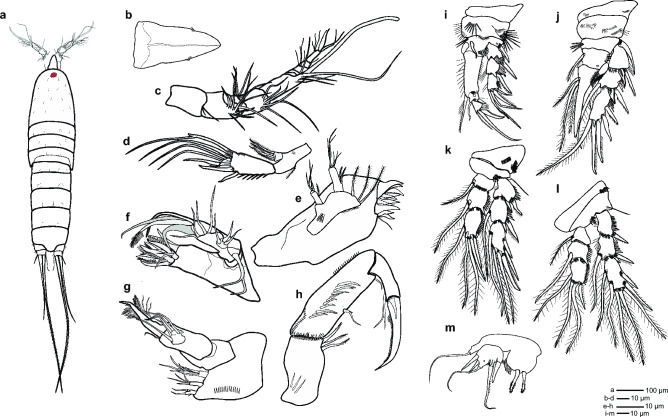
*Ikanecator primus* gen. et sp. nov, male morphology. (**a**) Habitus in dorsal view, (**b**) rostrum, (**c**) antennule, (**d**) antenna, (**e**) mandible, (**f**) maxillule, (**g**) maxilla, (**h**) maxilliped, (**i**) swimming leg 1, (**j**) swimming leg 2, (**k**) swimming leg 3, (**l**) swimming leg 4 and (**m**) swimming leg 5.

The average size of adult specimens (*n* = 20) is 994.5 ± 336.97 μm. This measurement was taken from the anterior margin of the rostrum to the posterior margin of the caudal rami. The body has a slender and cylindrical shape and blends together without a noticeable distinction between the prosome and urosome when viewed from above (Fig. 1, Fig. 2a–c and Fig. 3a). The body also lacks any surface decoration except for a hyaline frill on the urosomites. The cephalothorax is the widest portion of the body, and the anal segment is the narrowest.

The rostrum is a large, non-split structure that articulates with the cephalothorax (Fig. 2a, Fig. 3b and Supplementary Fig. 2). It tapers to a bell shape and features one pairs of sensory tiny setae located away from the center.

The antennule of male is made up of eleven segments, haplocer (Figs. 2d, Fig. 3c and Supplementary Fig. 2). All segments smooth, except for proximal and subdistal spinular rows on first segment. All setae are smooth in all segments. Armature formula: 1-(2), 2-(6), 3-(4), 4-(4), 5-(6), 6-(4 + aesthetasc), 7-(0), 8-(1), 9-(2), 10-(3), 11-(7).

The antennule of female has eight segments (Fig. 2a). The first four segments are much larger than the following four segments. There are two aesthetascs, where one is larger and arises from the anterior margin of the fourth segment, and the smaller one can be found on the last segment. Each segment carries from one to several smooth setae. Armature formula as follows: 1-(2), 2-(6), 3-(6), 4-(2 + aesthetasc), 5-(1), 6-(3), 7-(1), 8-(7).

The antenna can be divided into three distinct parts: the coxa, the exopod, and the endopod (Fig. 2d, Fig. 3d and Supplementary Fig. 3). The coxa is unprotected, whereas the exopod possesses three small spines on its surface and a single segment equipped with four setae that resemble feathers. Interestingly, there is only a single seta located opposite to the four feathered setae on the exopod. The endopod is armed with six geniculate elements, three spiniform setae, and five slender, pinnate setae. Additionally, the endopod is decorated with rows of small spinules of various sizes arranged along the inner margin and subdistal rim.

The mandible endopod is prominent with single two large, bicuspid teeth as well as numerous smaller teeth (Fig. 3e). It is also decorated with a single internal pinnate seta at the distal corner. The basis is furnished with various row of small spinules and possesses three setae on the inside. The exopod is comprised of two segments, where the first segment is longer than the second. The first segment also has three setae, while the second segment has just one apical seta. The endopod is formed of only a single segment, but is larger than the exopod, incorporating two lateral and six distal setae.

The maxillule is a simple structure (Figs. 2d and 3f). The coxa is ornamented with rows of small spinules, and the arthrite is well developed. The coxa also has eight spines at its distal margin, some of which have small setae. The exopod has four segments. The first segment has two setae, the second has three setae, and the third has four setae. The exopod also has four big setae at its distal margin.

The maxilla, a limb that has one large endopod, also has three endites (Figs. 2d and 3g). The endopod consists of four segments. The first two segments are plain, while the third has one small spine and it contain five setae of various sizes. The fourth segment has a single-haired pinnate setae with a large claw. Additionally, the maxilla syncoxa presents three endites. Of these, the proximal endite boasts two spines, the median endite four, and the distal endite ten, which are pinnate.

The maxilliped synoxa has several special features (Figs. 2d, 3h and Supplementary Fig. 3). It has three distal setae in the anterior surface and several rows of spinules are located close to basis. The eleven median setae in the middle portion of the basis are ornamented. On the anterior surface, there is both a subdistal median setae and a subdistal slender setae. The smaller rows are located at the beginning and ending parts of the posterior surface. The endopod consists of two segments. The first segment possesses two slender setae, which are pointy in shape. On the second segment, there is a large, sleek terminal claw that has a single spiniform setae attached to it.

The male's and female’s first leg’s coxa region are adorned with distinctive spines of different sizes arranged in rows on its frontal surface (Figs. 2d, 3i, Supplementary Fig. 4 and Supplementary Fig. 5). These spines number is reduced in females. Basis armed with an outer pinnate spine and an inner pinnate spine. The two spines, surrounded by different sized spinules, are located on the anterior part of the basis. The male’s basis has a curved extension at the top, while the female's does not have this particular extension. The exopod consists of three segments, with the EXP1 and EXP2 having a large outer pinnate spine with many smooth spines and the second exopod also having four outer four spines. EXP3 has two median smooth spines, two inner geniculate setae, several tiny spines. The endopod is made of three distinct segments. ENP1 is longer than ENP3 and is outfitted with an outer, single, pinnate spine as well as ten median spines. The length of ENP1 both sexes is for either equal to or less than the length of EXP. In addition, there is also an obvious hole in ENP2 near the ENP1. ENP2 stands out with a small hole and is distinct from other segments of the endopod. It carries two inner pinnate spine as well as an inner plumose setae and an outer medium setae. ENP3 is armed with an outer smooth spine that resembles a and tiny unguiforms, two median setae, one geniculate, and four median spines.

The second leg of the male is decorated with spinules arranged in rows on its anterior surface, with these spinules situated in three separate regions in praecoxa and coxa (Figs. 2d and 3j). The basis has outer pinnate spine, surrounded by smaller spinules. Additionally, four spines are located other side of basis. EXP1 has one outer pinnate spine and one median unguiform. This unique structural characteristic has been consistently found in all EXP1 and EXP2. In addition, EXP1 is encompassed by smaller spinules of various sizes. EXP2 is furnished with one outer spine, one unguiform, one inner plumose seta and numerous spinules. EXP3 boasts two outer, pinnate spines, two median spinulose spines, and one inner plumose seta with varying sizes of spinules and tiny unguiforms. The endopod has a two-segmented form with a unique shape that aids in the identification of sexual dimorphism. ENP1 is larger than that of the female, and it possesses one inner plusome seta, a small outer unguiform spine, and inner five spines. The first segment also stands out with a small hole. ENP2 is transformed into a large, strong, smooth, slowly curved, and tapering attenuation with two central ridges. It bears one medially directed knob close to the first segment, and at the base of this knob, three unequal length plumose setae arise.

**Figure 4:**
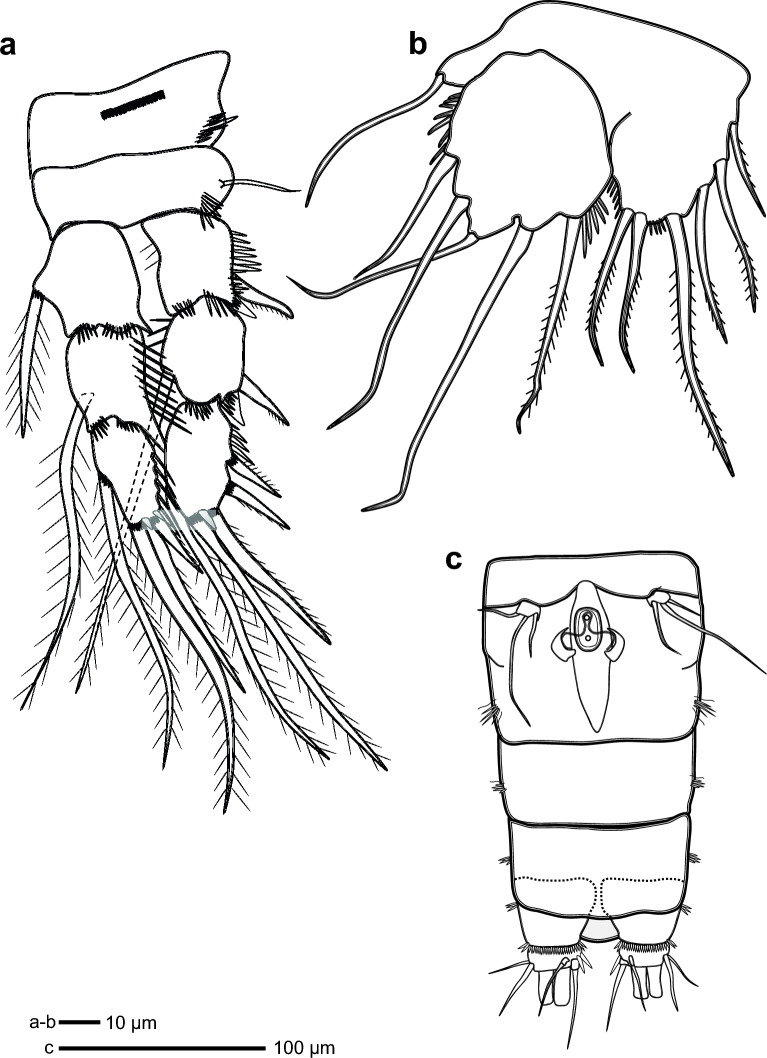
*Ikanecator primus* gen. et sp. nov, female. (**a**) Swimming leg 2, (**b**) swimming leg 5 and (**c**) genital field.

The median and anterior regions of the second leg of female coxa feature a line of spinules arranged in a single row (Fig. 4a and Supplementary Fig. 5). There is a slender seta found on the outer part of the basis and some small spines on the inner margin of the basis. The exopod, which is divided into three segments, possesses one outer pinnate spine and one unguiform in its first segment. Each of these spines has many spinules, which are slender projections that resemble spines. EXP2 has one outer pinnate spine, one unguiform, one inner plumose seta and many smaller spinules. The median spines in EXP3 include one spinulose spine and one plumose seta, while the outer spines are made up of two pinnate spines and one spinulose spine with tiny unguiforms and spinules. The endopod is made up of three segments. ENP1 and ENP2 each have a plumose inner seta with a small unguiform. ENP3 has one inner pectinate seta, two median plumose setae and a single outer spinulose spine.

The third leg of the male and the female are adorned with rows of spinules on its anterior surface in the coxa (Fig. 3k and Supplementary Fig. 5). The basis possesses multiple outer slender setae along with several spinules on the inner margin. The exopod is segmented into three parts. EXP1 carries one outer pinnate spine and one unguiform equipped with numerous spinules. EXP2 has one outer pinnate spine, a unguiform, a single inner plumose seta and bears many spinules. EXP3 possesses three spines: two pinnate and one spinulose, alongside two median plumose setae and one inner plumose seta that is characterized by numerous spinules and small unguiforms. The endopod is composed of three segments. ENP1 and ENP2 each possess an inner plumose seta adorned with spinules and a small size unguiform. ENP3 is armed with an outer pinnate spine, two median plumose setae, and two inner setae: one pectinate and one plumose, with inner rows of spinules and unguiforms.

The fourth leg of male and female coxa are ornamented with a several row of spinules on its anterior surface (Fig. 3l and Supplementary Fig. 5). Located medially between the exopod and endopod is the basis, which has a varying size of spinules. Also, basis has one outer slender seta. The exopod is three-segmented, with each segment adorned with spinules. EXP1 has one outer pinnate spine with numerous spinules and one unguiform, while EXP2 has one outer pinnate spines, one unguiform and an inner plumose setae. EXP3 has three outer spines: two pinnate and one spinulose, as well as two median elements: one spinulose spine and one plumose seta, and two inner setae: one pectinate and one plumose with spinules and small unguiforms. The endopod is also three-segmented, with ENP1 and ENP2 each having an inner plumose seta, one unguiform and ENP3 having an outer pinnate spine, a median plumose setae, and two inner plumose setae with spinules and unguiforms.

The male's fifth leg consists of an endopod and an exopod, with the endopod having a distinctive oval shape featuring two prominent inter spines (Figs. 2e and 3m). The setae are hair-like structures found at the foot of each spine. Additionally, the endopod has six spinules located on its inner margin. The outer seta is a slender bristle-like structure. The exopod is a segmented structure with a small hole in the middle. It is equipped with two smooth spines located on the outer part of the structure, a median smooth seta, and two inner elements. The inner elements consist of a smooth seta and a complex spinulose three-spine structure, also known as a comb-like structure.

The female’s fifth leg of exopod has oval-shaped bears five setae: three medium smooth, one thin smooth and one medium plumose setae with outer spinules and inner spinules (Figs. 2f, 4b and Supplementary Fig. 5). Baseoendopod has one outer smooth seta and bearing six plumose setae.

The sixth leg of the male and the female, which comes in the shape of a rectangular limb, carries a solitary tiny spine on the inside and two smooth bristles that run down the middle (Figs. 2e and 4c).

The posterior part of the urosome in both sexes exhibits a U-shaped projection in dorsal view (Fig. 2g). In ventral view of urosome in both gender show sexual dimorphism (Figs. 2f and 2h). The females urosome has five-segmented (Genital-double somite) the genital area contains a unique concave shape (Figs. 2f and 4c). This particular morphology has not been observed in male individuals. The male urosome has six-segmented.

### *Ikanecator primus* Zoral and Lajbner, gen. et sp. nov. molecular analyses

**Figure 5:**
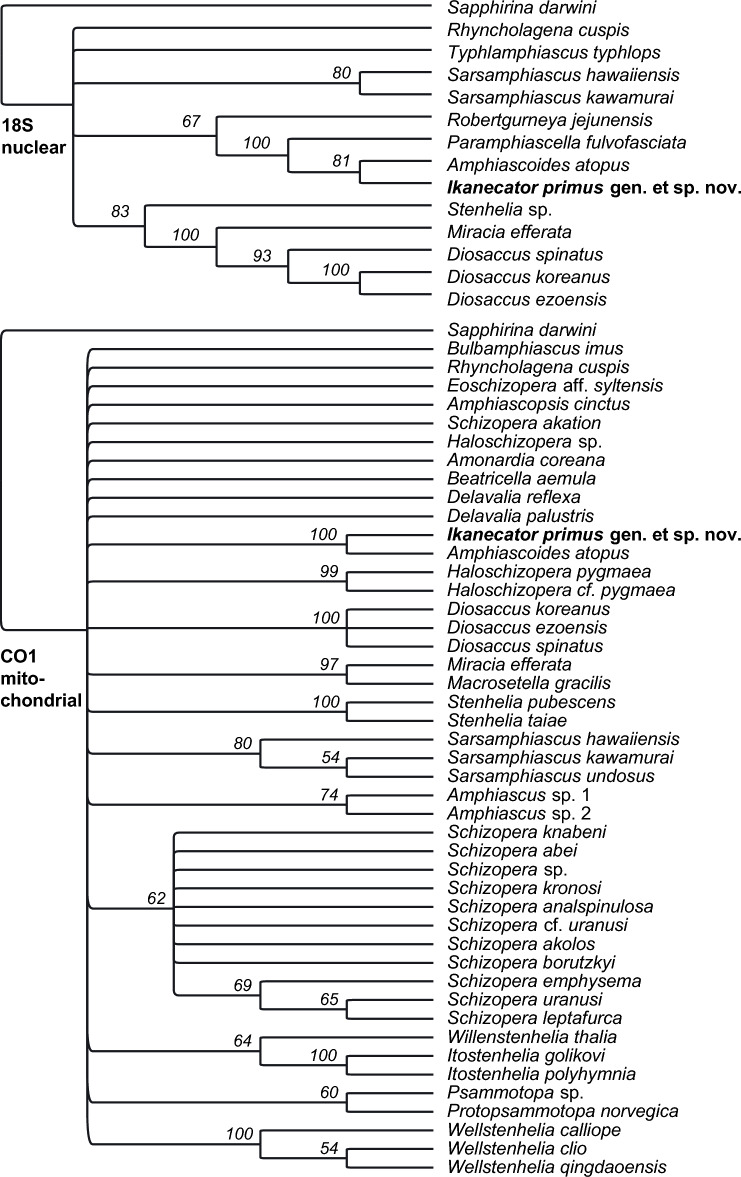
Consensus maximum likelihood phylogenetic tree of Miraciidae family rooted by *Sapphirina darwini* (Sapphirinidae family) based on a sequence of nuclear gene for 18S ribosomal subunit (above) and mitochondrial gene for cytochrome oxidase 1 (below) over 100 non-parametric bootstrap inferences. Branch labels show the consensus support (%) and branches with lower than 50% support are not shown.

Assembled and annotated DNA fragments as well as raw sequencing data are available in Genbank under project number PRJNA1065100 (see Supplementary Table 1 for details). Molecular phylogenetic analysis using nDNA sequence of 18S ribosomal subunit (PP196486) and mtDNA barcoding sequence of COI (PP163433) independently and unambiguously placed the parasitic copepod nearby genera Amphiascoides and Paramphiascella of the Miraciidae family, distant from all DNA sequences that we have found available in public databases Genbank and BOLD (Fig. 5, Supplementary Table 1). Reconstructed and annotated mitogenome (PP163433; Supplementary Fig. 6) appears distinct from related genus *Amphiascoides* (NCBI blastn returns 76% identity on matching parts of *Amphiascoides atopus*, NC_023783.1; 91% query cover).

We have created 407,085 scaffolds of N50 length 2173 bp (Supplementary Data 1, sheet Quast) and 90% of scaffolds were unclassified by Kraken software that classifies preferentially bacterial reads, presumably originating in copepod. Around 7% was classified as of microbial origin (Supplementary Data 1, sheet Kraken2), where Pseudomonadota (Proteobacteria in old nomenclature) phylum was the most abundant (Supplementary Data 1, sheet taxonomy_all). Most of the annotated scaffolds belonged to hypothetical proteins or to house-keeping genes. Arthropoda scaffolds with highest coverage (over 300) were assigned functions such as DNA polymerase, cytochrome oxidase subunit I and other enzymes involved in oxidative phosphorylation. We have used arthropodal scaffolds to assess copepod genome completeness with BUSCO, which was 72.6%. Total repetitive bases masked were 59,047,466 bp, which is 14.35% of the genome. Most common were simple repeats (1.90%), followed by Class I retroelement repeats (1.35%). Class II repeats comprised of DNA transposons take up 0.57% of genome (Supplementary Data 1, sheet repeatmasker). Carbohydrate active enzymes were found on 2,431 scaffolds, of which 426 can be assigned with high confidence to Eukaryote and 302 to Arthropoda (Supplementary Data 1, sheet taxonomy_all). We were interested to find enzymes involved in degradation of mucopolysaccharides of squid egg capsules, as this copepod species is grazing on them. Indeed, we have found some of the enzymes involved in degradation of squid egg capsules (Table 1) in both Eukaryotic and Arthropoda scaffolds. We have found genes for enzymes involved in mucopolysaccharides degradation on 23 scaffolds belonging to Arthropoda. This showcases the potential of copepod for degradation of squid egg casing. There were also 24 scaffolds belonging to bacteria that encodes for the genes involved in mucopolysaccharides degradation. We have found 28 CAZy families on both prokaryotic and arthropodal scaffolds (Supplementary Data 1, sheet bac_arthro_cazy.), 21 CAZy families only in Arthropoda and 111 CAZy families only in bacteria. Bacterial specific enzyme activities were degradation of peptidoglycan (bacterial cell wall) and pullulan (polymer produced by fungus *Aureobasidium pullulans*). Interestingly, we have found cellulose degradation enzymes (GH7—mostly found in fungi, GH5_24, cellobiohydrolase) in this copepod genome, which were also present in the genomes of other copepods *Tigriopus californicus* and *Eurytemora affinis*. Genes encoding enzymes involved in degradation of polyphenols (laccase, peroxidase), chitin (chitinase, lysozyme), hemicellulose (endo-mannanase) and cellulose (cellobiohydrolase) were found in both prokaryotic and arthropodal scaffolds (Supplementary Data 1, sheet cazy_all and sheet bac_arthro_cazy).

**Table 1:** Enzymes potentially involved in degradation of mucopolysaccharides (based on Wraith 2013) found in cephalopod egg capsules. Most of these enzymes do not have representative family in Carbohydrate Active EnZymes database (www.cazy.org). Cazy families of glycoside hydrolases that were identified either in bacterial or arthropodal scaffolds are showed below.

EC number	Enzyme function	GH families detected in arthropodal scaffolds	GH families detected in bacterial scaffolds
2.3.1.3	Acetyl CoA glucosamine n-acetyl transferase	–	–
3.1.6.12	Galactosamine-4-sulfatase	–	–
3.1.6.13	Iduronate-2-sulfatase	–	–
3.1.6.14	N-acetyl-glucosamine-6-sulfatase	–	–
3.1.6.4	Galactose-6-sulfatase	–	–
3.10.1.1	Heparan-n-sulfatase	–	
3.2.1.23	β-Galactosidase	GH1, GH2, GH5, GH16	GH1, GH2, GH3, GH16, GH35, GH39, GH42, GH147
3.2.1.31	β-Glucuronidase	GH1, GH2, GH79	GH2, GH79
3.2.1.35	Hyaluronidase	GH16, GH84	GH16, GH84
3.2.1.50	N-acetyl-glucosaminidase	GH89	GH89
3.2.1.76	α-l-iduronidase	–	GH39

### Antiparasitic effects of PAA-product in vitro

The cessation or lack of movement in adult copepods were examined after the addition of each specific dose including 250 μl/L (35 mg/L PAA and 15 mg/L H_2_O_2_), 500 μl/L (70 mg/L PAA and 30 mg/L H_2_O_2_) and 1000 μl/L (140 mg/L PAA and 60 mg/L H_2_O_2_) of PAA-product within specific time range (Supplementary Table 3). The copepod stopped moving completely within 2 min after exposure to 500 and 1000 μl/L PAA-product, starting with an evident reduction in movement. In addition, the first death of an adult exposed to 250 μl/L was observed in 47.6 min, while the last adult died in 138.2 min. Interestingly, no mortality was observed in copepods kept at 125 μl/L (17.5 mg/L PAA and 7.5 mg/L H_2_O_2_) of PAA-product for 2 weeks.

No nauplii were observed at any treatment groups with 250, 500, and 1000 μl/L of PAA product. Nauplii were detected between day 4 to day 6 in copepod eggs treated with 125 μl/L PAA-product and controls (Table 2).

**Table 2:** Time to *Ikanecator primus* gen. et sp. nov., nauplii hatch from egg which was exposed to different concentrations of PAA-product for 2 min in vitro (n = 3 replicates; 20 adult female parasites with eggs per replicate).

	Dose of PAA-product (μl/l)	Nauplii hatched in 18 days	The day the first Nauplii detected
In vitro replicate 1	Water control (1)	YES	Day 5
	Water control (2)	YES	Day 4
	125	YES	Day 4
	250	NO	N/A
	500	NO	N/A
	1000	NO	N/A
In vitro replicate 2	Water control (1)	YES	Day 5
	Water control (2)	YES	Day 6
	125	YES	Day 5
	250	NO	N/A
	500	NO	N/A
	1000	NO	N/A
In vitro replicate 3	Water control (1)	YES	Day 5
	Water control (2)	YES	Day 5
	125	YES	Day 5
	250	NO	N/A
	500	NO	N/A
	1000	NO	N/A

### Acute toxicity test on squid embryos before PAA-product immersion treatment application

Survival of squid embryos is presented in Table 3, following the hatching of all viable eggs that were treated with PAA-product for 2 min. When a toxic amount of PAA-product was applied, the egg color started to change from transparent to white during the incubation period within the next 3 days and embryonal development ceased. The toxicity effect was dependent upon concentration (Table 3). The concentration of 1661.13 ± 68.13 μl/L (232.55 mg/L PAA and 99.66 mg/L H_2_O_2_) PAA-product was toxic to 50% of embryos at 2 min.

**Table 3:** Acute toxicity test (LC_10_, LC_50_ and LC_90_) of the PAA-product in *Sepioteuthis lessoniana* sp.1 (n = 3 replicates; 10 eggs per replicate).

	Duration of treatment (min)	LC_10_ (μl/l)	LC_50_ (μl/l)	LC_90_ (μl/l)
PAA-product	2	1290.65 ± 40.89	1661.13 ± 68.13	2148.19 ± 126.86

### Immersion treatment with PAA-product

No parasite was observed after being treated with PAA-product at concentration of 500 μl/L (70 mg/L PAA and 30 mg/L H_2_O_2_) and 1000 μl/L (140 mg/L PAA and 60 mg/L H_2_O_2_) in all squid eggs including *S. lessoniana* sp.1, *S. lessoniana* sp.2 and *S. lessoniana* sp.3 (Table 4). The rate of prevalence was significantly changed with PAA-product at concentration 500 and 1000 μl/L compared the control (*p* < 0.05). Although the parasite population at 250 μl/L (35 mg/L PAA and 15 mg/L H_2_O_2_) of PAA-product apparently decreased, at least 1 adult parasite was detected in all eggs. At 125 μl/L (17.5 mg/L PAA and 7.5 mg/L H_2_O_2_) of PAA-product, there was no apparent decrease in the parasite population compared to the control.

**Table 4:** Prevalence of adult *Ikanecator primus* gen. et sp. nov., infection in oval squid eggs following immersion treatment of PAA-product (8 replicates in total: *Sepioteuthis lessoniana* sp.1 n = 6 replicates; 30 eggs per replicate; *S. lessoniana* sp.2 n = 4 replicates; 30 eggs per replicate; *S. lessoniana* sp.3 n = 3 replicates; 30 eggs per replicate).

Host	Dose of PAA-product (μl/l)	Duration of PAA-product treatment (min)	Prevalence of adult *Ikanecator primus* (%)
*Sepioteuthis lessoniana* sp.1	Water control	2	100
	125	2	100
	250	2	100
	500	2	0
	1000	2	0
*Sepioteuthis lessoniana* sp.2	Water control	2	100
	125	2	100
	250	2	100
	500	2	0
	1000	2	0
*Sepioteuthis lessoniana* sp.3	Water control	2	100
	125	2	100
	250	2	100
	500	2	0
	1000	2	0

### Hatching rate of squid eggs and hatchling size

**Figure 6:**
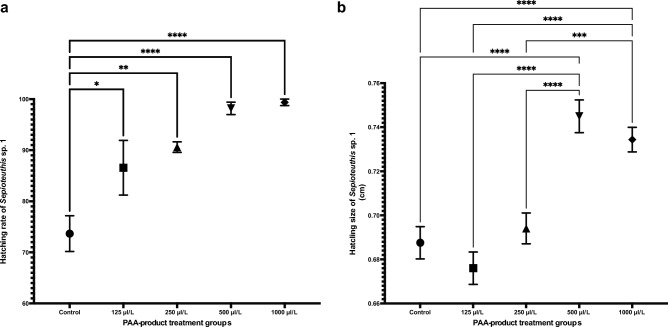
The positive effects on the survival rate and growth of *Sepioteuthis lessoniana* sp.1 hatchlings due to PAA-product treatment. Hatching rate of *S. lessoniana* sp.1 from each experimental treatment group (*n* = 6 replicates; 30 eggs per replicate). (**a**) All treatment groups were compared to the control with significant differences shown as: *p < 0.05, ** *p* < 0.005, **** *p* < 0.0001. (**b**) Hatchling size (cm) as the total mantle length within 24 h post hatching of *S. lessoniana* sp.1 which was exposed to different concentrations of PAA-product for 2 min by immersion treatment (*n* = 6 replicates; 30 eggs per replicate). 500 and 1000 μl/l of PAA-product groups were compared to 125 and 250 μl/l of PAA-product and the control with significant differences shown as: *** *p* < 0.001, **** *p* < 0.0001.

**Figure 7:**
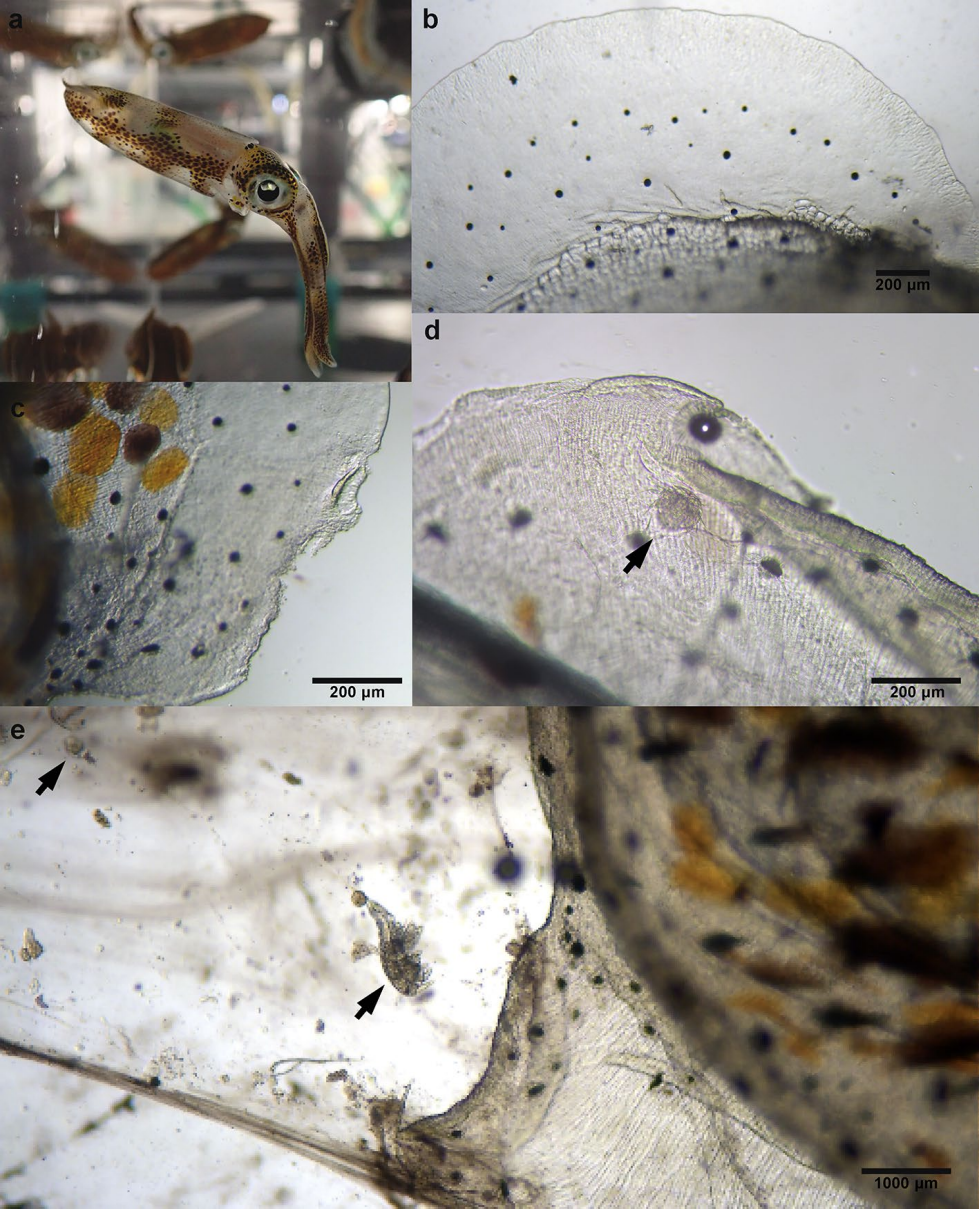
*Ikanecator primus* gen. et sp. nov., poses a threat to the health of *Sepioteuthis lessoniana* sp.1. (**a**) Following 500 and 1000 μl/l of PAA-product treatment, squids in all treatment groups did not show any signs of abnormality, including loss of appetite, cachexia, and lethargy for four weeks after hatching. (**b**) No fin damage was evident in the group of squid treated with PAA-product at a concentration of 500 and 1000 µl/L (see Supplementary Video 3). (**c**) Squid hatchlings in the control group show injuries to their fins and fin erosion. (**d**) The black arrow shows *I. primus*, nauplius infecting a 1-day-old squid hatchling (see Supplementary Video 4). (**e**) The black arrows show *I. primus*, nauplius and adult infecting a 1-day-old squid hatchling (see Supplementary Video 5). Erosion of the fins is severe and leads to an increase in mucus secretion from the affected area (see Supplementary Video 6) (The squid shown in Supplementary Video 5 is the same individual depicted in Supplementary Video 6, specifically showing its fin).

The PAA-product application improved the hatching rate (%) of squid eggs in all treatment groups compared to the control (Fig. 6a). The clinical signs of abnormality such as loss of appetite, cachexia and lethargy were not observed in squids in all treatment groups held for 4 weeks after hatching (Fig. 7a). No fin damage was observed in squid in the groups treated with 500 and 1000 µl/L PAA-product (Fig. 7b and Supplementary Video 3). Small group of red-squid treated with 500 µl/L PAA-product were kept until reproduction. The life cycle of the PAA-treated red-squid in captivity has been closed in OIST Marine Science Station at day 215 post-hatching (January 23rd, 2024). Eggs were treated with 500 µl/L PAA-product again and resulting juveniles did not show any obvious abnormality.

The squid hatchlings in the control group were infected with nauplii. The nauplii attached to the fins of the squid hatchlings (Fig. 7d and Supplementary Video 4). The hatchlings experienced injuries to their fins and fin erosion caused by nauplii and adult copepods (Fig. 7c–e, Supplementary Video 5 and Supplementary Video 6). The squid shown in Supplementary Video 5 is the same individual depicted in Supplementary Video 6, specifically showing its fin.

The results of the experiment showed that the PAA-product application at 500 and 1000 μl/L significantly affected and increased hatchling size in the treatment groups compared to 125 and 250 μl/L of PAA-product and the controls (*p* < 0.0001; Fig. 6b). The mean total mantle length of the control group was 0.6875 ± 0.0804 cm, while the PAA-product treated with concentrations of 500 and 1000 μl/L groups were 0.7433 ± 0.0780 cm and 0.7343 ± 0.0682 cm respectively.

## Discussion

Cephalopod aquaculture is still in its infancy when compared to the well-established technologies of fish and shrimp farming, but the rapid cephalopod growth and high edible content make them promising organisms for aquaculture^2,3^. Considering that up to 50% of the production loss in fish culture is caused by diseases, there is a need for research, especially in the field of disease management, for squid farming to reach its full potential^31^. The purpose of this study was to increase squid hatching rate and survival. Our results uncovered parasitic copepod as the culprit pathogen responsible for reduced hatching rate in our experimental facility. This study also demonstrates that peracetic acid solution is effective in treating parasitic copepod infections in squid eggs and the peracetic acid treatment increased squid hatching rate and survival.

There have been limited studies on copepods infecting cephalopods^32,33^. In rare cases, copepods have been found in the skin of adult cephalopods collected from the wild during gross pathology examinations^33,34^. Additionally, copepods have been also found in wild-collected cephalopod eggs, and it has been speculated that this may be a problem for the health of the eggs or hatchlings^35,36^. Specifically, there are no available data on copepod infection discovered in squid eggs under aquaculture conditions.

Our morphological examination and molecular-genomic results have shown that a parasitic copepod new to science, *Ikanecator primus* gen. et sp. nov., from the Miraciidae family do infect squid eggs. This newly discovered genus shares some similar morphological features with genus of *Amphiascoides* (45 species), *Paramphiascella* (28 species) and *Robertgurneya* (31 species) that have previously been documented by World Register of Marine Species^37^. Our morphological examination revealed that the *I. primus* possesses distinctive structural characteristics that set it apart from another species and genera. For example, the length of ENP1 in *I. primus* is either equal to or less than the length of EXP. According to genus description of *Amphiascoides* and *Paramphiascella* ENP1 always reach far beyond EXP3^38,39^. The male *I. primus* antennule has haplocer, 11-segmented whereas the male in *Robertgurneya* genus has haplocer, 10-segmented^39,40^.

Our study shows that doses of 500 μl/L (70 mg/L PAA and 30 mg/L H_2_O_2_) and above of PAA-product demonstrated antiparasitic activity against these copepods within 2 min. At a lower dose of 250 μl/L PAA-product (35 mg/L PAA and 15 mg/L H_2_O_2_), PAA-product was also effective but took more than 2 h, hence this dose is not recommended for practical and short immersion applications on infected squid eggs with copepods. Previous studies have shown that PAA is dose-dependent and can inhibit the in vitro growth of fish bacteria, including *Flavobacterium columnare* (1–8 mg/L of PAA)^41^, *Aeromonas salmonicida* (0.1–2 mg/L of PAA)^42^, *Yersinia ruckeri* (0.1–2 mg/L of PAA)^42^, and *Vibrio fischeri* (less than 2 mg/L of PAA)^43,44^. PAA (0.2–8 mg/L) has also been effective against *Saprolegnia* sp., a fungus infection in *Ictalurus punctatus* (egg) and *Salmo salar* (fry size)^41,45^. PAA (15 mg/L) treatment eliminates *Ichthyophthirius multifiliis*, a single-celled ciliated protozoan species common parasite known as white spot disease, in all stages including theronts, tomonts, and cysts^46–49^. We demonstrated that PAA-product application has antiparasitic effects against metazoans, namely copepods belonging to the Miraciidae family. The effective dose of PAA-product (70 mg/L PAA and 30 mg/L H_2_O_2_) against parasitic copepod infection is higher than that required to eliminate bacterial, fungal, and protozoan infections.

The development of copepods includes nauplii and adult stages. Copepod eggs hatch as free-living nauplius larvae while still attached to the adult female’s paired egg strings^50^. Proper understanding of the copepod life cycle is critical for disease management. So, we focused on the effect of PAA-product on copepod eggs to disrupt the pathogen's life cycle^14^. Our study found that a 250 μl/L of PAA-product (35 mg/L PAA and 15 mg/L H_2_O_2_) exposure for 2 min inhibited nauplii from egg strings in vitro. Previous research has reported similar antiparasitic results but only using H_2_O_2_ in high doses. For example, when 1750 mg/L of H_2_O_2_ was tested in vitro on the eggs of *Lepeophtheirus salmonis* and *Caligus rogercresseyi*, it was observed that development of parasitic copepod larvae ceased from these eggs exposed to H_2_O_2_^51,52^. Through our findings, we can deduce that PAA is essential in repressing parasitic copepod larvae development and H_2_O_2_ is inconsequential in this process. Going forward, it is imperative to further investigate the impact of PAA on different species of parasitic copepod larvae to establish the optimal concentrations for efficient inhibition.

PAA is eco-friendly and it does not leave behind persistent residues. However, PAA use in aquatic environments has been known to harm non-target organisms such as mollusks and fish. For instance, PAA has been found to be toxic to certain mollusk species, with 24-h LC_50_ values ranging between 0.28 mg/L for *Mytilus edulis* embryo and *Crassostrea gigas* embryo^53^. To assess the viability of immersion treatment with a PAA-product on squid embryos, we conducted toxicity assays during incubation. Our results showed that when exposed to a 2-min dose of the PAA-product, the LC_50_ was 1661.13 ± 68.13 μl/L (232.55 mg/L PAA and 99.66 mg/L H_2_O_2_). We observed that the dead eggs changed color from transparent to white during the incubation period. Additionally, embryo development stopped in the egg. The severity of our PAA treatment application could be higher due its combination with H_2_O_2_. Some research found that a positive correlation: by associating PAA with H_2_O_2_, they confirmed that this association had a more toxic effect on Crustacea, *Daphnia magna* and fish species (*Cyprinus carpio*, *Carassius auratus*, *Ctenopharyngodon idella*, *Cyclopterus lumpus*, *Danio rerio*, *Ictalurus punctatus*, *Micropterus salmoides*, *Notemigonus crysoleucas*, *Oncorhynchus mykiss*, *Pomoxis nigromaculatus*, *Sander vitreus*) to the point that the 24-h LC_50_ value decreased accordingly^53,54,56–58^.

Our clinical trial found that immersing squid eggs in the PAA-product of 500 μl/L (70 mg/L PAA and 30 mg/L H_2_O_2_) for 2 min was the safest and most effective method of eliminating parasitic copepod infections, resulting in a 0% prevalence of parasitic copepod on the surface of the eggs. This immersion treatment method could be used in squid aquaculture to combat parasitic copepod infections. In addition, this study is the first to use PAA with H_2_O_2_ treatment on cephalopod eggs; previous studies applied only H_2_O_2_ on fry or adult fish species to treat parasitic copepod infections in aquaculture^59,60^.

The application of PAA-product at concentrations of 500 μl/L (70 mg/L PAA and 30 mg/L H_2_O_2_) and 1000 μl/L (140 mg/L PAA and 60 mg/L H_2_O_2_) on eggs resulted in squid hatching rates of 98.18% and 99.35%, respectively. At these concentrations, the treatment was successful in getting rid of copepods. However, when the concentration of PAA-product was only 125 μl/L (17.5 mg/L PAA and 7.5 mg/L H_2_O_2_) and 250 μl/L (35 mg/L PAA and 15 mg/L H_2_O_2_), the intensity of parasite infection was reduced, but some copepods were still present, which likely affected squid hatching rate (86.54% and 90.59% respectively). Nevertheless, these results were still promising compared to controls with average hatching rate just 73.66%.

Our findings also showed that treatment groups exposed to 500 μl/L and 1000 μl/L PAA-product had significantly larger hatchling sizes compared to the groups exposed to 125 μl/L and 250 μl/L PAA-product, as well as the control (*p* < 0.05). We assume the *I. primus* elimination by PAA reduces incidence of squid premature hatching and thus increases the hatchling size. Squids that hatched from the PAA-treated squid eggs, at a concentration of 500 µl/L, displayed no obvious abnormal behaviors even in subsequent generation. This suggests that the PAA-product treatment doesn’t have any strong negative adverse effect on squid in the long term. Nonetheless, further studies should examine the PAA toxicology in more detail and on molecular level. Although we might anticipate a comparable effect in all cephalopods, additional studies are needed to establish effective doses for other cephalopod species.

The strong positive effect of PAA-product treatment on squid egg is mainly due to the elimination of physical damage caused by the grazing activity of copepods. We found genes for many enzymes that might be involved in degradation of squid egg capsules that were assigned to Arthropoda, thus possibly originating in copepod genome (Table 1). Such enzymes were also found on prokaryotic scaffolds with more diverse representation of glycoside hydrolase families than in case of arthropodal scaffolds (Table 1). These secreting enzymes enable the bacterial community to digest the surface of squid egg capsule and reduce its permeability manipulating oxygen diffusion. Previous studies showed that oxygen plays a critical role in cephalopod embryonal development and low oxygen levels inside cephalopod egg masses can delay or kill developing cephalopod embryosy^61–64^. Furthermore, it is possible that some of these enzymes are secreted also by bacterial symbionts in the copepod digestive tract. Additional studies will be required to assess the complex nature of this host-parasite interaction^65,66^. Most likely, the disruption of these potentially harmful microbial communities represents another substantial benefit of the PAA-product treatment.

Our research has discovered a new copepod species, *Ikanecator primus* gen et sp. nov. on squid eggs. This species is threatening squid eggs as a parasite, which represents a challenge in the field of cephalopod culture. Fortunately, we demonstrated that PAA-products can be used as therapeutic agents against copepod infections on squid eggs. Safe doses can be applied by immersion treatment to increase the hatching rate, survival rate and size of squid hatchlings. This novel treatment method shows great potential as a safe and effective way to control parasitic copepod infection in cephalopod culture.

### Supplementary Information

Supplementary Data.

Supplementary Figures.

Supplementary Tables.

Supplementary Videos.

## Data Availability

The Genbank database, which is the United States National Library of Medicine’s National Center for Biotechnology Information, contains the annotated database of all publicly available DNA sequences. Specifically, the raw reads can be found in Genbank under the accession numbers provided (SRR27696250, SRR27696251 and PRJNA1065100). The DNA sequences coding for the small subunit ribosomal RNA (18S rRNA) and the cytochrome oxidase I (COI) containing mitogenomic sequence have been submitted and are available under accession numbers PP196486 and PP163433 respectively (Supplementary Table 1). These were used for phylogenetic tree reconstructions (Fig. 5).

## References

[1] FAO (2018). The state of world fisheries and aquaculture.

[2] Satjarak J, Thongprajukaew K, Kaewtapee C, Rodjan P, Preedaphol K (2022). Morphological characteristics and nutritive value of wild and cultured bigfin reef squid (*Sepioteuthis lessoniana)*. J. Food Compos. Anal..

[3] Vidal E.A.G, Villanueva R., Andrade J.P., Gleadall I.G., Iglesias J., Koueta N., Rosas, Segawa S., Grasse B., Franco-Santos R., Albertin C., Caamal-Monsreal C., Chimal M., Edsinger-Gonzales E., Gallardo P., Le Pabic C., Pascual C., Roumbedakis K., Wood J., 2014. Chapter One - Cephalopod culture: current status of main biological models and research priorities, E.A.G. Vidal (Ed.), Advances in Marine Biology, Academic Press (2014), pp. 1–98. 10.1016/B978-0-12-800287-2.00001-9 24880794

[4] Pierce GJ, Portela J (2014). Fisheries Production and Market Demand, Cephalopod Culture (eds Iglesias J. et al.) Springer. Dordrecht.

[5] Nabhitabhata J., Ikeda I., 2014. *Sepioteuthis lessoniana*. In: Iglesias J, Fuentes L, Villanueva R (eds) Cephalopod culture. Dordrecht: Springer Science & Business Media, 315–347 pp. 10.1007/978-94-017-8648-5_12

[6] Cheng SH, Anderson FE, Bergman A, Mahardika GN, Muchlisin ZA, Dang BT, Calumpong HP, Mohamed KS, Sasikumar G, Venkatesan V, Barber PH (2014). Molecular evidence for co-occurring cryptic lineages within the *Sepioteuthis cf. lessoniana* species complex in the Indian and Indo-West Pacific Oceans. Hydrobiologia.

[7] Jereb P., Roper C.F.E., Cephalopods of the World. An Annotated and Illustrated Catalogue of Cephalopod Species Known to Date. Myopsid and Oegopsid Squids Vol. 2 (FAO, 2010).

[8] Imai H., Aoki M., Genetic diversity and genetic heterogeneity of bigfin reef squid “*Sepioteuthis lessoniana*” species complex in northwestern Pacific Ocean. in Analysis of Genetic Variation in Animals (Caliskan, M. ed). pp. 151–166 (2012).

[9] Nakajima R, Lajbner Z, Kuba MJ, Gutnick T, Iglesias TL, Asada K, Nishibayashi T, Miller J (2022). Squid adjust their body color according to substrate. Sci. Rep..

[10] Izuka T, Segawa S, Okutani T, Numachi K (1994). Evidence on the existence of three species in the oval squid Sepioteuthis lessoniana complex in Ishigaki Island Okinawa southwestern Japan by isozyme analyses. Venus (Jpn. J. Malacol.).

[11] Izuka T (1996). Biochemical study of the population heterogeneity and distribution of the oval squid *Sepioteuthis lessoniana* complex in southwestern Japan. Am. Malac. Bull..

[12] Noga E.J., Fish Disease: Diagnosis and Treatment, 2nd Edition Wiley-Blackwell, Iowa, USA, (2010).

[13] Bass D, Rueckert S, Stern R, Cleary AC, Taylor JD, Ward GM, Huys R (2021). Parasites, pathogens, and other symbionts of copepods. Trends Parasitol..

[14] Lester R.J.G., Hayward C.J., Phylum Arthropoda. P.T.K. Woo (Ed.), Fish Diseases and Disorders, Volume 1: Protozoan and Metazoan Infections, CAB International, pp. 466–565 (2006).

[15] Costello MJ (2006). Ecology of sea lice parasitic on farmed and wild fish. Trends Parasitol..

[16] Costello MJ (2009). The global economic cost of sea lice to the salmonid farming industry. J. Fish Dis..

[17] Tarrant AM, Nilsson B, Winding-Hansen B (2019). Molecular physiology of copepods—from biomarkers to transcriptomes and back again Comp. Biochem. Physiol. D.

[18] Llewellyn MS, Leadbeater S, Garcia C, Sylvain F-E, Custodio M, Ang KP, Powell F, Carvalho GR, Creer S, Elliot J, Derome N (2017). Parasitism perturbs the mucosal microbiome of Atlantic Salmon. Sci. Rep..

[19] Abu-Elala NM, Attia MM, Abd-Elsalam RM, Gamal A, Younis NA (2021). Peracetic acid treatment of Ichthyophthirius multifiliis (Ciliophora: Ichthyophthiriidae) and Trichodina spp. reduces the infection by Aeromonas hydrophila and improves survival in Nile tilapia (Oreochromis niloticus). Aquaculture.

[20] European Chemicals Agency, Regulation (EU) No 528/2012 Concerning the Making Available on the Market and Use of Biocidal Products. Assessment Report for peracetic acid (2015).

[21] Kitis M (2004). Disinfection of wastewater with peracetic acid: A review. Environ. Int..

[22] Lieke T, Meinelt T, Hoseinifar SH, Pan B, Straus DL, Steinberg CEW (2020). Sustainable aquaculture requires environmental-friendly treatment strategies for fish diseases. Rev. Aquac..

[23] Mota VC, Eggen ML, Lazado CC (2022). Acute dose-response exposure of a peracetic acid-based disinfectant to Atlantic salmon parr reared in recirculating aquaculture systems. Aquaculture.

[24] Smail DA, Grant R, Simpson D, Bain N, Hastings TS (2004). Disinfectants against cultured Infectious Salmon Anaemia (ISA) virus: the virucidal effect of three iodophors, chloramine T, chlorine dioxide and peracetic acid/hydrogen peroxide/acetic acid mixture. Aquaculture.

[25] Lieke T, Meinelt T, Hoseinifar SH, Pan B, Straus DL, Steinberg CEW (2019). Sustainable aquaculture requires environmental-friendly treatment strategies for fish diseases. Rev. Aquac..

[26] Teitge F, Peppler C, Steinhagen D, Jung-Schroers V (2020). Effect of disinfection with peracetic acid on the microbial community of a seawater aquaculture recirculation system for Pacific white shrimp (*Litopenaeus vannamei*). J. Fish Dis..

[27] Segawa S (1987). Life history of the oval squid, *Sepioteuthis lessoniana* in Kominato and adjacent waters central Honshu, Japan. J. Tokyo Univ. Fish..

[28] Manni M, Berkeley MR, Seppey M, Simão FA, Zdobnov EM (2021). BUSCO update: novel and streamlined workflows along with broader and deeper phylogenetic coverage for scoring of eukaryotic, prokaryotic, and viral genomes. Mol. Biol. Evol..

[29] Stamatakis A (2014). RAxML Version 8: A tool for phylogenetic analysis and post-analysis of large phylogenies. Bioinformatics.

[30] Hamilton MA, Russo RC, Thursto NV (1977). Trimed Spearman-Karber method for estimating medial lethal concentrations in toxicity bioassays. Environ. Sci. Technol..

[31] Assefa A, Abunna F (2018). Maintenance of fish health in aquaculture: review of epidemiological approaches for prevention and control of infectious disease of fish. Vet. Med. Int.

[32] Avdeev G.V., Amplipedicola pectinatus gen. et sp. n. (Copepoda, Harpacticoida, Tisbidae), a parasite of octopuses in the Bering Sea. Crustaceana 83, pp. 1363–1370 (2010). http://www.jstor.org/stable/41038645

[33] Tedesco P, Bevilacqua S, Fiorito G, Terlizzi A (2020). Global patterns of parasite diversity in cephalopods. Sci. Rep..

[34] Pascual S., Abollo E., Mladineo I., Gestal C., Metazoa and Related Diseases In: Gestal C, Pascual S, Guerra A, Fiorito G, Vieites JM (eds) Handbook of pathogens and diseases in cephalopods. Springer Nature, Switzerland, AG, Open access. 169–179 (2019).

[35] Hanlon R.T., Maintenance, rearing, and culture of teuthoid and sepioid squids. Gilbert D.L., Adelman W.J., Arnold J.M. (eds) Squid as experimental animals, (pp. 35–62). Springer, Boston, MA (1990). 10.1007/978-1-4899-2489-6

[36] De Silva-Dávila R, Avendaño-Ibarra R, Palomares-García R, Markaida U (2019). First records of an egg mass and a paralarva of *Thysanoteuthis rhombus* (Cephalopoda: Thysanoteuthidae) in the northeastern tropical Pacific. Pac. Sci..

[37] WoRMS Editorial Board, World Register of Marine Species. Available from https://www.marinespecies.org at VLIZ. Accessed 2023-12-04 (2023). 10.14284/170

[38] Marcotte BM (1974). Two new harpacticoid copepods from the North Adriatic and a revision of the genus Paramphiascella. Zool. J. Linn. Soc..

[39] Gómez S, Corgosinho PHC, Rivera-Sánchez KI (2021). Proposal of new genera and species of the subfamily diosaccinae (Copepoda: Harpacticoida: Miraciidae). Eur. J. Taxon..

[40] Yeom J, Lee W (2022). A New Species of the Genus Robertgurneya Apostolov & Marinov, 1988 (Copepoda: Harpacticoida: Miraciidae) from a Sublittoral Zone of Jeju Island, Korea. Diversity.

[41] Marchand PA, Phan TM, Straus DL, Farmer BD, Stuber A, Meinelt T (2012). Reduction of *in vitro* growth in *Flavobacterium columnare* and *Saprolegnia parasitica* by products containing peracetic acid. Aquac. Res..

[42] Meinelt T, Phan TM, Behrens S, Wienke A, Pedersen LF, Liu D, Straus DL (2015). Growth inhibition of *Aeromonas salmonicida* and *Yersinia ruckeri* by disinfectants containing peracetic acid. Dis. Aquat. Org..

[43] Antonelli M, Mezzanotte V, Panouillères M (2009). Assessment of peracetic acid disinfected effluents by microbiotests. Environ. Sci. Technol..

[44] Chhetri RK, Thornberg D, Berner J, Garmstad R, Öjstedt U, Sharma AK, Andersen HR (2014). Chemical disinfection of combined sewer overflow waters using performic acid or peracetic acids. Sci. Total Environ..

[45] DiCocco A, May T, Crouse C, Marancik D, Phuntumart V, Ghosh S, Beligala GU, Redman N, Murray M, Fischer G, Summerfelt S, Good C (2021). Reducing mortality associated with opportunistic infections in Atlantic salmon *Salmo salar* fry using hydrogen peroxide and peracetic acid. Aquac. Res..

[46] Meinelt T, Matzke S, Stubert A, Pietrock M, Wienke A, Mitchell AJ, Strauss D (2009). Toxicity of peracetic acid (PAA) to tomonts of *Ichthyophthirius multifiliis*. Dis. Aquat. Org..

[47] Straus DL, Meinelt T (2009). Acute toxicity of peracetic acid (PAA) formulations to *Ichthyophthirius multifiliis* theronts. Parasitol. Res..

[48] Picon-Camacho SM, Marcos-Lopez M, Beljean A, Debeaume S, Shinn AP (2012). *In vitro* assessment of the chemotherapeutic action of a specific hydrogen peroxide, peracetic, acetic, and peroctanoic acid-based formulation against the free-living stages of *Ichthyophthirius multifiliis* (Ciliophora). Parasitol. Res..

[49] Sudova E, Straus DL, Wienke A, Meinelt T (2010). Evaluation of continuous 4-day exposure to peracetic acid as a treatment for *Ichthyophthirius multifiliis*. Parasitol. Res..

[50] Bravo S (2010). The reproductive output of sea lice *Caligus rogercresseyi* under controlled conditions. Exp. Parasitol..

[51] Bravo S, Silva MT, Agusti C, Sambra K, Horsberg TE (2015). The effect of chemotherapeutic drugs used to control sea lice on the hatching viability of egg strings from *Caligus rogercressey*. Aquaculture.

[52] McAndrew KJ, Sommerville C, Wootten R, Bron JE (1998). The effects of hydrogen peroxide on different life-cycle stages of the salmon louse, *Lepeophtheirus salmonis* (Krøyer, 1837). J. Fish Dis..

[53] Henao LD, Turolla A, Antonelli M (2018). Disinfection by-products formation and ecotoxicological effects of effluents treated with peracetic acid: A review. Chemosphere.

[54] Liu D, Straus DL, Pedersen L, Meinelt T (2015). Comparison of the toxicity of Wofasteril peracetic acid formulations E400, E250, and Lspez to *Daphnia magna*, with emphasis on the effect of hydrogen peroxide. N. Am. J. Aquacult..

[55] Liu D, Pedersen LF, Straus DL, Kloas W, Meinelt T (2017). Alternative prophylaxis/disinfection in aquaculture - adaptable stress induced by peracetic acid at low concentration and its application strategy in RAS. Aquaculture..

[56] Straus DL, Meinelt T, Farmer BD, Beck BH (2012). Acute toxicity and histopathology of channel catfish fry exposed to peracetic acid. Aquaculture.

[57] Straus D.L., Ledbetter C.K., Farmer B.D., Meinelt T., Pedersen L.F., Acute toxicity of peracetic acid to various fish species 17th International Conference on Diseases of Fish and Shellfish, European Association of Fish Pathologists, Las Palmas, 188 (2015).

[58] Ytteborg E, Lazado CC, Noble C, Hansen RI, Johansen LH (2023). The skin mucosal barrier of lumpfish (Cyclopterus lumpus L.) is weakened by exposure to potential aquaculture production-related stressors. J. Fish Biol..

[59] Burridge L, Weis JS, Cabello F, Pizarro J, Bostick K (2010). Chemical use in salmon aquaculture: A review of current practices and possible environmental effects. Aquaculture.

[60] Overton K, Samsing F, Oppedal F, Dalvin S, Stien LH, Dempster T (2018). The use and effects of hydrogen peroxide on salmon lice and post-smolt Atlantic salmon. Aquaculture.

[61] Seymour RS (1994). Oxygen diffusion through the jelly capsules of amphibian eggs. Isr. J. Zool..

[62] Cronin ER, Seymour RS (2000). Respiration of the eggs of the giant cuttlefish *Sepia apama*. Mar. Biol..

[63] Gutowska MA, Melzner F (2009). Abiotic conditions in cephalopod (*Sepia officinalis*) eggs: embryonic development at low pH and high *p*CO_2_. Mar. Biol..

[64] Vidal EAG, von Boletzky S, Iglesias J, Fuentes L, Villanueva R (2014). *Loligo vulgaris* and *Doryteuthis opalescens*. Dordrecht.

[65] Baden T (2023). Cephalopod-omics: Emerging fields and technologies in cephalopod biology. Integr.
Comp. Biol..

[66] Rosa, R. *et al.* Past, present, and future trends in octopus research. Octopus Biology and Ecology. Academic Press, 421–454. 10.1016/B978-0-12-820639-3.00010-8.

