# Supplementary material for: Peracetic acid treatment of squid eggs infected with parasitic copepod (Ikanecator primus gen. et sp. nov.)
Source: Sci Rep. 2024 Jun 24;14:14513. doi: 10.1038/s41598-024-65290-z (PMC11196259; doi:10.1038/s41598-024-65290-z)
Supplement: Supplementary file 2 — Supplementary Figures. [file 41598_2024_65290_MOESM2_ESM.docx]

**Supplementary Figure 1.** *Ikanecator primus* gen. et sp. nov. nauplius infecting *Sepioteuthis lessoniana* sp.1 egg.





**Supplementary Figure 2.** *Ikanecator primus* gen. et sp. nov., paratype, male. Rostrum and antennule in front view (NMP P6E 5487, the National Museum of the Czech Republic).

**Supplementary Figure 3.** *Ikanecator primus* gen. et sp. nov., paratype, male. Antenna (a), maxilliped (mxp) (NMP P6E 5486, the National Museum of the Czech Republic).


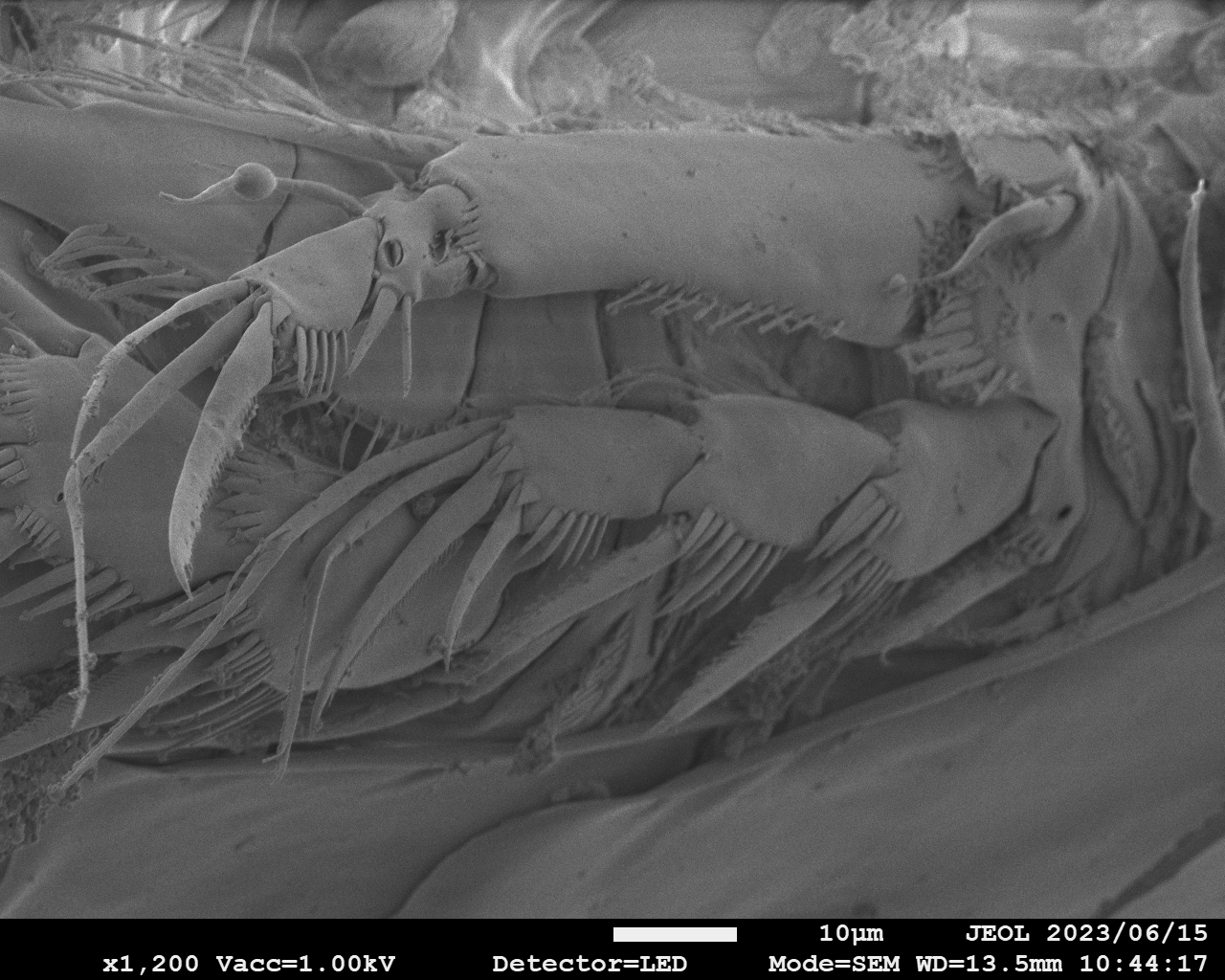


**Supplementary Figure 4.** *Ikanecator primus* gen. et sp. nov., paratype, male. Swimming leg 1 (NMP P6E 5486, the National Museum of the Czech Republic).

**Supplementary Figure 5.** *Ikanecator primus* gen. et sp. nov., paratype, female. Swimming leg 1 (p1), swimming leg 2 (p2), swimming leg 3 (p3), swimming leg 4 (p4), swimming leg 5 (p5) (NMP P6E 5486, the National Museum of the Czech Republic).

**Supplementary Figure 6.** Mitochondrial DNA of *Ikanecator primus* gen. et sp. nov.
