# Supplementary material for: Peracetic acid treatment of squid eggs infected with parasitic copepod (Ikanecator primus gen. et sp. nov.)
Source: Sci Rep. 2024 Jun 24;14:14513. doi: 10.1038/s41598-024-65290-z (PMC11196259; doi:10.1038/s41598-024-65290-z)
Supplement: Supplementary file 3 — Supplementary Tables. [file 41598_2024_65290_MOESM3_ESM.docx]

**Supplementary Table 1.** GenBank numbers of DNA sequences used in this study.

| Gene marker | Species name | Accession no. | Reference |
| --- | --- | --- | --- |
| 18S | *Amphiascoides atopus* | [KC815328](http://www.ncbi.nlm.nih.gov/nuccore/KC815328) | S Gomez et al., 2013, unpublished data |
| 18S | *Diosaccus ezoensis* | [KR048740](http://www.ncbi.nlm.nih.gov/nuccore/KR048740) | SY Baek, UW Hwang, 2015, unpublished data |
| 18S | *Diosaccus koreanus* | [MT002900](http://www.ncbi.nlm.nih.gov/nuccore/MT002900) | [Lim et al. (2020](https://doi.org/10.3897/zookeys.927.49042)) |
| 18S | *Diosaccus spinatus* | [EU380290](http://www.ncbi.nlm.nih.gov/nuccore/EU380290) | [Huys et al. (2009)](https://doi.org/10.1016/j.ympev.2008.12.007) |
| 18S | *Ikanecator primus* gen. et sp. nov. | PP196486 | This paper |
| 18S | *Miracia efferata* | [EU380294](http://www.ncbi.nlm.nih.gov/nuccore/EU380294) | [Huys et al. (2009)](https://doi.org/10.1016/j.ympev.2008.12.007) |
| 18S | *Paramphiascella fulvofasciata* | [EU380293](http://www.ncbi.nlm.nih.gov/nuccore/EU380293) | [Huys et al. (2009)](https://doi.org/10.1016/j.ympev.2008.12.007) |
| 18S | *Rhyncholagena cuspis* | [OR257803](http://www.ncbi.nlm.nih.gov/nuccore/OR257803) | [Yeom & Lee (2023)](https://doi.org/10.3897/zookeys.1180.109288) |
| 18S | *Robertgourneya jejuensis* | [OP798781](http://www.ncbi.nlm.nih.gov/nuccore/OR798781) | [Yeom & Lee (2022)](https://doi.org/10.3390/d14121127) |
| 18S | *Sapphirina darwinii* | [GU969173](http://www.ncbi.nlm.nih.gov/nuccore/GU969173) | M Wang, S Sun, 2010, unpublished data |
| 18S | *Sarsamphiascus hawaiiensis* | [MN496456](http://www.ncbi.nlm.nih.gov/nuccore/MN496456) | [Yeom & Lee (2020)](https://doi.org/10.7717/peerj.8506) |
| 18S | *Sarsamphiascus kawamurai* | [MN541391](http://www.ncbi.nlm.nih.gov/nuccore/MN541391) | [Yeom & Lee (2020)](https://doi.org/10.7717/peerj.8506) |
| 18S | *Stenhelia* sp. | [EU380291](http://www.ncbi.nlm.nih.gov/nuccore/EU380291) | [Huys et al. (2009)](https://doi.org/10.1016/j.ympev.2008.12.007) |
| 18S | *Typhlamphiascus typhlops* | [EU380292](http://www.ncbi.nlm.nih.gov/nuccore/EU380292) | [Huys et al. (2009)](https://doi.org/10.1016/j.ympev.2008.12.007) |
| COI | *Amonardia coreana* | [KT030279](http://www.ncbi.nlm.nih.gov/nuccore/KT030279) | SY Baek, UW Hwang, 2015, unpublished data |
| COI | *Amphiascoides atopus* | [NC023783](http://www.ncbi.nlm.nih.gov/nuccore/NC023783) | [Easton et al. (2014)](https://doi.org/10.1016%2Fj.gene.2013.12.053) |
| COI | *Amphiascopsis cinctus* | [MH670487](http://www.ncbi.nlm.nih.gov/nuccore/MH670487) | [Rossel & Arbizu (2019)](https://doi.org/10.1038%2Fs41598-019-45718-7) |
| COI | *Amphiascus* sp. 1 | [KX714909](http://www.ncbi.nlm.nih.gov/nuccore/KX714909) | [Gollner et al. (2016)](https://doi.org/10.1371%2Fjournal.pone.0163776) |
| COI | *Amphiascus* sp. 2 | [KX714910](http://www.ncbi.nlm.nih.gov/nuccore/KX714910) | [Gollner et al. (2016)](https://doi.org/10.1371%2Fjournal.pone.0163776) |
| COI | *Beatricella aemula* | [MH670532](http://www.ncbi.nlm.nih.gov/nuccore/MH670532) | [Rossel & Arbizu (2019)](https://doi.org/10.1038%2Fs41598-019-45718-7) |
| COI | *Bulbamphiascus imus* | [MH670539](http://www.ncbi.nlm.nih.gov/nuccore/MH670539) | [Rossel & Arbizu (2019)](https://doi.org/10.1038%2Fs41598-019-45718-7) |
| COI | *Delavalia palustris* | [MH976544](http://www.ncbi.nlm.nih.gov/nuccore/MH976544) | [Rossel & Arbizu (2019)](https://doi.org/10.1038%2Fs41598-019-45718-7) |
| COI | *Delavalia reflexa* | [MH976545](http://www.ncbi.nlm.nih.gov/nuccore/MH976545) | [Rossel & Arbizu (2019)](https://doi.org/10.1038%2Fs41598-019-45718-7) |
| COI | *Diosaccus ezoensis* | [KR049013](http://www.ncbi.nlm.nih.gov/nuccore/KR049013) | SY Baek, UW Hwang, 2015, unpublished data |
| COI | *Diosaccus koreanus* | [MN996281](http://www.ncbi.nlm.nih.gov/nuccore/MN996281) | [Lim et al. (2020](https://doi.org/10.3897/zookeys.927.49042)) |
| COI | *Diosaccus spinatus* | [HQ966504](http://www.ncbi.nlm.nih.gov/nuccore/HQ966504) | [A Telfer, N Jeffery, 2010, iBOL](http://boldsystems.org/index.php/Public_RecordView?processid=NJCGS307-10) |
| COI | *Eoschizopera* sp. aff. *syltensis* | [MH976580](http://www.ncbi.nlm.nih.gov/nuccore/MH976580) | [Rossel & Arbizu (2019)](https://doi.org/10.1038%2Fs41598-019-45718-7) |
| COI | *Haloschizopera pygmaea* | [MH976598](http://www.ncbi.nlm.nih.gov/nuccore/MH976598) | [Rossel & Arbizu (2019)](https://doi.org/10.1038%2Fs41598-019-45718-7) |
| COI | *Haloschizopera* sp. | [MH976603](http://www.ncbi.nlm.nih.gov/nuccore/MH976603) | [Rossel & Arbizu (2019)](https://doi.org/10.1038%2Fs41598-019-45718-7) |
| COI | *Haloschizopera* sp. cf. pygmaea | [MH976604](http://www.ncbi.nlm.nih.gov/nuccore/MH976604) | [Rossel & Arbizu (2019)](https://doi.org/10.1038%2Fs41598-019-45718-7) |
| COI | *Ikanecator primus* gen. et sp. nov. | PP163433 | This paper |
| COI | *Itostenhelia golikovi* | [KF524864](http://www.ncbi.nlm.nih.gov/nuccore/KF524864) | [Karanovic, Kim & Lee (2014)](https://doi.org/10.3897%2Fzookeys.411.7346) |
| COI | *Itostenhelia polyhymnia* | [KF524838](http://www.ncbi.nlm.nih.gov/nuccore/KF524838) | [Karanovic, Kim & Lee (2014)](https://doi.org/10.3897%2Fzookeys.411.7346) |
| COI | *Macrosetella gracilis* | [MG742365](http://www.ncbi.nlm.nih.gov/nuccore/MG742365) | P Santhanam, et al., 2017, unpublished data |
| COI | *Miracia efferata* | [GU171350](http://www.ncbi.nlm.nih.gov/nuccore/GU171350) | [Bucklin et al. (2010)](https://doi.org/10.1016%2Fj.dsr2.2010.09.025) |
| COI | *Protopsammotopa norvegica* | [MK037095](http://www.ncbi.nlm.nih.gov/nuccore/MK037095) | [Rossel & Arbizu (2019)](https://doi.org/10.1038%2Fs41598-019-45718-7) |
| COI | *Psamotopa sp.* | [MK037062](http://www.ncbi.nlm.nih.gov/nuccore/MK037062) | [Rossel & Arbizu (2019)](https://doi.org/10.1038%2Fs41598-019-45718-7) |
| COI | *Rhyncholagena cuspis* | [OR252612](http://www.ncbi.nlm.nih.gov/nuccore/OR252612) | [Yeom & Lee (2023)](https://doi.org/10.3897/zookeys.1180.109288) |
| COI | *Sapphirina darwinii* | [HM045389](http://www.ncbi.nlm.nih.gov/nuccore/HM045389) | S Sun, M Wang, C Li, 2016, unpublished data |
| COI | *Sarsamphiascus hawaiiensis* | [MN507530](http://www.ncbi.nlm.nih.gov/nuccore/MN507530) | [Yeom & Lee (2020)](https://doi.org/10.7717/peerj.8506) |
| COI | *Sarsamphiascus kawamurai* | [MN542379](http://www.ncbi.nlm.nih.gov/nuccore/MN542379) | [Yeom & Lee (2020)](https://doi.org/10.7717/peerj.8506) |
| COI | *Sarsamphiascus undosus* | [MH242965](http://www.ncbi.nlm.nih.gov/nuccore/MH242965) | M Leray & G Paulay, 2018, unpublished data |
| COI | *Schizopera abei* | [KP867870](http://www.ncbi.nlm.nih.gov/nuccore/KP867870) | [Karanovic, Kim & Grygier (2015)](http://dx.doi.org/10.1080/00222933.2015.1028112) |
| COI | *Schizopera akation* | [JQ390560](http://www.ncbi.nlm.nih.gov/nuccore/JQ390560) | [Karanovic & Cooper (2012)](https://doi.org/10.1071%2FIS11027) |
| COI | *Schizopera akolos* | [JQ390584](http://www.ncbi.nlm.nih.gov/nuccore/JQ390584) | [Karanovic & Cooper (2012)](https://doi.org/10.1071%2FIS11027) |
| COI | *Schizopera analspinulosa* | [JQ390588](http://www.ncbi.nlm.nih.gov/nuccore/JQ390588) | [Karanovic & Cooper (2012)](https://doi.org/10.1071%2FIS11027) |
| COI | *Schizopera borutzkyi* | [OP830326](http://www.ncbi.nlm.nih.gov/nuccore/OP830326) | C Marshall, et al., 2023, unpublished data |
| COI | *Schizopera cf. uranusi* | [JQ390571](http://www.ncbi.nlm.nih.gov/nuccore/JQ390571) | [Karanovic & Cooper (2012)](https://doi.org/10.1071%2FIS11027) |
| COI | *Schizopera emphysema* | [JQ390558](http://www.ncbi.nlm.nih.gov/nuccore/JQ390558) | [Karanovic & Cooper (2012)](https://doi.org/10.1071%2FIS11027) |
| COI | *Schizopera knabeni* | [KF667527](http://www.ncbi.nlm.nih.gov/nuccore/KF667527) | [Easton et al. (2014)](https://doi.org/10.1016%2Fj.gene.2013.12.053) |
| COI | *Schizopera kronosi* | [JQ390567](http://www.ncbi.nlm.nih.gov/nuccore/JQ390567) | [Karanovic & Cooper (2012)](https://doi.org/10.1071%2FIS11027) |
| COI | *Schizopera leptafurca* | [JQ390590](http://www.ncbi.nlm.nih.gov/nuccore/JQ390590) | [Karanovic & Cooper (2012)](https://doi.org/10.1071%2FIS11027) |
| COI | *Schizopera sp.* | [MW021221](http://www.ncbi.nlm.nih.gov/nuccore/MW021221) | K Cullen & M Harvey, 2020, unpublished data |
| COI | *Schizopera uranusi* | [JQ390561](http://www.ncbi.nlm.nih.gov/nuccore/JQ390561) | [Karanovic & Cooper (2012)](https://doi.org/10.1071%2FIS11027) |
| COI | *Stenhelia pubescens* | [KF524870](http://www.ncbi.nlm.nih.gov/nuccore/KF524870) | [Karanovic, Kim & Lee (2014)](https://doi.org/10.3897%2Fzookeys.411.7346) |
| COI | *Stenhelia taiae* | [KF524885](http://www.ncbi.nlm.nih.gov/nuccore/KF524885) | [Karanovic, Kim & Lee (2014)](https://doi.org/10.3897%2Fzookeys.411.7346) |
| COI | *Wellstenhelia calliope* | [KF524872](http://www.ncbi.nlm.nih.gov/nuccore/KF524872) | [Karanovic, Kim & Lee (2014)](https://doi.org/10.3897%2Fzookeys.411.7346) |
| COI | *Wellstenhelia clio* | [KF524873](http://www.ncbi.nlm.nih.gov/nuccore/KF524873) | [Karanovic, Kim & Lee (2014)](https://doi.org/10.3897%2Fzookeys.411.7346) |
| COI | *Wellstenhelia qingdaoensis* | [KF524874](http://www.ncbi.nlm.nih.gov/nuccore/KF524874) | [Karanovic, Kim & Lee (2014)](https://doi.org/10.3897%2Fzookeys.411.7346) |
| COI | *Willenstenhelia thalia* | [KF524882](http://www.ncbi.nlm.nih.gov/nuccore/KF524882) | [Karanovic, Kim & Lee (2014)](https://doi.org/10.3897%2Fzookeys.411.7346) |
| Mitogenome | *Ikanecator primus* gen. et sp. nov. | PP163433 | This paper |
| Raw reads | *Ikanecator primus* gen. et sp. nov. | SRR27696250  SRR27696251 PRJNA1065100 | This paper |

**Supplementary Table 2.** Armature formula of P1-P5.

|  | EXP | ENP |
| --- | --- | --- |
| P1 | 1-0;1-0;2-2 | 1-0-0;2-1-0;0-2-1 |
| P2 ♀ / ♂ | 1-1-0;1-1-1;3-0-2 | 1-1-0;1-1-0;1-2-1/dimorphic |
| P3 | 1-1-0-0;1-1-1-0;2-0-3-1 | 1-1-0-0;1-1-0-0;2-0-2-1 |
| P4 | 1-1-0-0-0;1-1-1-0-0;2-2-0-2-1 | 1-1-0-0;1-1-0-0;1-0-2-1 |
| P5 ♀ / ♂ | 3-1-1/2-1-2 | 6-1/2-1 |

**Supplementary Table 3.** *In vitro* effects of different PAA-product concentrations for 2 minutes on the survival of adult *Ikanecator primus* gen. et sp. nov. (n = 3 replicates; 20 adult parasites per replicate).

| Dose of PAA-product (μl/l) | Mortality (%) at  2 min | Time to Death (min) | |
| --- | --- | --- | --- |
|  |  | The first death of adult copepod | The last death of an adult copepod |
| Water control | 0 | >20160 | >20160 |
| 125 | 0 | >20160 | >20160 |
| 250 | 0 | 47.6 | 138.2 |
| 500 | 100 | 0.53 | 1.65 |
| 1000 | 100 | 0.06 | 0.38 |
